# Antidiscrimination Interventions, Political Ads on Transgender Rights, and Public Opinion: Results From Two Survey Experiments on Adults in the United States

**DOI:** 10.3389/fpsyg.2021.729322

**Published:** 2021-08-19

**Authors:** Andrew R. Flores, Donald P. Haider-Markel, Daniel C. Lewis, Patrick R. Miller, Jami K. Taylor

**Affiliations:** ^1^Department of Government, School of Public Affairs, American University, Washington, DC, United States; ^2^School of Law, The Williams Institute, University of California, Los Angeles, Los Angeles, CA, United States; ^3^Department of Political Science, University of Kansas, Lawrence, KS, United States; ^4^Department of Political Science and International Relations, Siena College, Loudonville, NY, United States; ^5^Department of Political Science and Public Administration, The University of Toledo, Toledo, OH, United States

**Keywords:** transgender rights, public opinion, prejudice reduction, survey experiment, media effects, backfire effects

## Abstract

Political advertisements can shift attitudes and behaviors to become more exclusionary toward social out-groups. However, people who engage in an antidiscrimination exercise in the context of an experiment may respond differently to such ads. What interventions might foster inclusive attitudes in the presence of political communications about social policy issues like transgender rights? We examined two scalable antidiscrimination exercises commonly used in applied settings: describing a personal narrative of discrimination and perspective-taking. We then showed people political ads that are favorable or opposed to transgender rights to determine whether those interventions moderate how receptive people are to the messages. Relying on two demographically representative survey experiments of adults in the United States (study 1 *N* = 1,291; study 2 *N* = 1,587), we found that personal recollections of discriminatory experiences did not reduce exclusionary attitudes, but perspective-taking had some effects, particularly among those who fully complied with the exercise. However, both studies revealed potential backfire effects; recalling a discriminatory experience induced negative attitudes among a subset of the participants, and participants who refused to perspective-take when prompted also held more negative attitudes. Importantly, political ads favorable toward transgender rights consistently resulted in more positive attitudes toward transgender people. Future work needs to carefully examine heterogeneous responses and resistance to antidiscrimination interventions and examine what particular aspects of the political ads induced the attitude change.

## Introduction

Social movement organizations, like those that promote transgender rights, spend considerable effort communicating about issues while working to change prejudicial attitudes (Tadlock, 2014; Flores, 2019). In trying to reduce prejudicial attitudes, individual level interventions can sometimes work (e.g., Paluck, 2009; Facchini et al., 2016; Harrison and Michelson, 2019). One strategy is to encourage people to recall a time when they have been discriminated against in order to make them aware of multiple forms of bias (Gorski and Pothini, 2018; Kalla and Broockman, 2021). Another strategy is to ask participants to take the perspective of others (Broockman and Kalla, 2016; Gorski and Pothini, 2018). Unfortunately, these approaches tend to be resource intensive and time consuming. In addition, both can invite resistance or backlash by putting individuals in a threatened position (Mikula, 1993; Vorauer and Sasaki, 2009; Vorauer et al., 2009; Todd and Galinsky, 2014; Mooijman and Stern, 2016; but see Stone et al., 2011). In this research, comprised of two survey experiments, we investigate how shorter recall (Study 1) and briefer perspective-taking interventions (Study 2) can affect attitudes about transgender rights, particularly regarding bathroom access. To increase the applicability of our research, we also exposed participants to competing massages about transgender rights with real-world advertisements about gender identity protections in public accommodations and restroom use. Our intent was to find effective but less resource intensive interventions that would reduce prejudices toward transgender people and transgender rights.

The results were mixed. Personally recalling discrimination did not increase support for attitudes about transgender people’s bathroom use based on their gender or general attitudes toward transgender people and rights (Study 1). On the contrary, some members of dominant groups became more antagonistic to transgender people and rights when performing the recollection exercises, and they were less persuaded by pro-transgender ads and sometimes more receptive to anti-transgender ads. In the entire study sample and for members of marginalized groups, they were unaffected by personal recollections, but they were persuaded by both anti- and pro-transgender ads. People who engaged with a slightly more intense perspective-taking exercise had higher levels of support for transgender rights, were slightly more receptive to pro-transgender ads, and were slightly less receptive to anti-transgender ads (Study 2). However, people who refused to perform the perspective-taking exercise had background characteristics leading them to resist the exercise and held more negative views toward transgender rights. Our findings suggested that antidiscrimination exercises induced differential responses among participants, and that the effect of the advertisements was conditioned by individual interventions. We concluded that personally recalling discriminatory experiences is potentially counter-productive by inducing status threat among dominant group members, perspective-taking might be promising but further research is needed to examine potential backfires and resistance, and advertisements in favor of transgender rights had consistently positive effects. Since antidiscrimination exercises are widely used in applied contexts (Gorski and Pothini, 2018; see also, Broockman and Kalla, 2016; Adida et al., 2018; Kalla and Broockman, 2020, 2021), our studies underscore that careful attention to complying with the exercises and potential backfires are important considerations when attempting to reduce prejudices.

## Political Ads and Ad Receptivity

Political advertisements may cause people to evaluate candidates, public policies, and groups differently (Gilens, 1999; Mendelberg, 2001; Brewer, 2002). Ads provide different meanings and portrayals of policy problems (Gamson, 1992). People who are exposed to ads may update their beliefs with new information about that issue and prioritize the concerns that the ads raise (Nelson and Oxley, 1999). Messages in ads can emphasize a variety of core values (Brewer, 2008) and put those values into conflict with one another (Alvarez and Brehm, 2002). When exposed to these messages, people may change their expressed attitude.

Political communications tend to have their largest effects on issues of low importance (Lecheler et al., 2009). In the United States, LGBT rights have relatively low public salience (Lindaman and Haider-Markel, 2002) and transgender rights has only recently emerged as a cleavage in American politics (Taylor et al., 2018). This may mean that people lack information about transgender people and transgender rights (Flores, 2015; Flores et al., 2018), so attitudes may not be as crystalized (e.g., Tesler, 2015). The potential for ads to influence opinions is likely high. As such, ads in opposition to transgender inclusive accommodations policies should reduce support for transgender people and rights (*H1*), and ads in favor of transgender inclusive accommodations policies will increase support for transgender people and rights (*H2*).

### Ad Receptivity and Cognitive States

Yet, the effectiveness of ads is not only influenced by the ad content, but also by the cognitive state of the individual receiving this content. Individuals become far more receptive to arguments that emphasize a loss as opposed to a gain (Tversky and Kahneman, 1981). People who are in cognitive state of anxiety may be more receptive to loss messages (Arceneaux, 2012; see also Albertson and Gadarian, 2015). Thus, a person’s cognitive state may interact with the advertisement’s information, emphasized values, and primed group identities (Druckman and Lupia, 2016).

A person’s cognitive state, however, is malleable. It may be altered by antidiscrimination exercises that facilitate awareness of overt and subtle biases that may characterize their attitudes toward groups, particularly stigmatized groups (e.g., Paluck, 2009; Facchini et al., 2016; Harrison and Michelson, 2019). These exercises are designed to increase awareness of inequality and close the perceived differences between the self and other (Kalla and Broockman, 2021). In this way, prejudicial attitudes and behaviors can be reduced. As a result, individuals may be in a cognitive state that is more willing to be affirming of out-groups, and these exercises should induce a cognitive state that would make an individual more aware of biases in the world around them. In so doing, they may be more favorable to the rights of transgender people (*H3*), less receptive to anti-transgender ads (*H4*), and more receptive to pro-transgender ads that favor the transgender rights (*H5*). This would be consistent with the findings of Broockman and Kalla (2016), who found that the durable attitude change from canvassing persisted after anti-transgender advertisements were shown to research participants in follow-up online experiments.

However, antidiscrimination exercises are sensitive processes, because acknowledging one’s own internalized biases may make one vulnerable or even defensive (Mikula, 1993; Vorauer and Sasaki, 2009; Vorauer et al., 2009; Mooijman and Stern, 2016; but see Stone et al., 2011). For example, some interventions on LGBT prejudice have the potential to backfire (Cramwinckel et al., 2018). This is usually why these exercises take place in a highly controlled or tailored environment. Yet, these controlled environments may not be feasible for larger scale advocacy efforts to reduce prejudice in the general population, and it is not clear whether approaches can be effectively scaled up to the mass level (e.g., Adida et al., 2018). While it is anticipated that these exercises will have their intended effects, we suspect that subgroups within the broader population may negatively respond to them. In our first study, we anticipate that describing personal experiences of discrimination may threaten the status of some members of the dominant group and provoke resentment. In our second study, we anticipate that some group members may refuse to perspective-take. In Study 1, we suspect that these subgroups will adversely respond by becoming more opposed to the rights of transgender people (*H6*), more receptive to anti-transgender ads (*H7*), and less receptive to pro-transgender ads (*H8*). We make descriptive observations about resisters in Study 2.

## Study 1: Recalling Personal Experiences of Discrimination

The aim of Study 1 is to investigate the effects of performing an exercise where participants recall their own personal experiences of discrimination. This is related to an “analogic perspective-taking” exercise, when after people tell their own experiences, they are prompted to connect such experiences to other groups (Kalla and Broockman, 2021). A difference in our exercises was that this final step was not encouraged. While recalling personal experiences of discrimination is intended to make a person aware of numerous forms of bias, it also elicits emotional responses consistent with status threat (Mikula et al., 1998). Status threat increases the salience of certain social identities (Lalonde and Cameron, 2013). In turn, these salient social identities condition ad effectiveness (Diamond, 2020), and a person’s relative group position should condition the effects of status threat (Nadler et al., 2009).

When an individual perceives that their group status is threatened, their response to such threats can vary. Importantly, one’s relative group position prior to status threat is an important factor in considering how individuals respond. Social groups array themselves in hierarchies (Schneider and Ingram, 1993). There are members of groups with dominant characteristics and groups with marginal characteristics (e.g., race, gender, and sexual orientation), and they vary in their access to social, economic, and political resources (Goffman, 1963; Schneider and Ingram, 1993). Social psychologists have examined both “high status” and “low status” groups in a minimal-group paradigm and in real-life contexts to show that individuals perceive the relative position of their group membership to that of others (Nadler et al., 2009). Relative group positions are then socially understood and psychologically internalized in one’s self-concept and group attachments.

Psychological responses to status threats are conditioned by one’s relative group position in society (Mullen et al., 1992). Nadler et al. (2009) show that response to status threats are conditioned by the stability of group hierarchies. Since 2016 in the United States, for example, there has been discussion of white group identity consciousness in response to economic and demographic status threats (Mutz, 2018; Jardina, 2019). The perceived precarious status of dominant group members in contemporary American politics is likely one reason racial attitudes more strongly predicted presidential vote choice in 2016 than in previous elections (Mutz, 2018; Sides et al., 2019). Thus, responses to status threat may be stronger among dominant group-members in the contemporary United States (Jardina, 2019). When facing threats, members of dominant groups with a precarious status should hold more favorable attitudes toward their own group and less favorable attitudes toward other groups.

However, an alternative response to status threat is for members to engage in helping behaviors to out-groups that restores the perceived position of their own group’s status. Helping behaviors, while documented among both dominant and marginal group members, are motivated by similar causes under different conditions. Under threatening conditions when the dominant group’s position is stable and when marginal groups have unstable positions, dominant group members may engage in helping behavior, further securing the position of their group as dominant while helping those from marginal groups (Nadler et al., 2009). In addition, marginal group members may be inclined to engage in out-group helping in what is known as defensive helping (Nadler et al., 2009). Defensive helping occurs as a response to a status threat because helping members of other marginal groups recovers a person’s own status within their group and remedies an injury to their self-esteem (Nadler et al., 2009). It occurs when there is a psychological need to reinforce a positive image and helping would not further subordinate a group.

Thus, we expect status threats to both dominant and marginal group members to condition their receptivity to ads in favor of or in opposition to other marginalized groups. However, we anticipate that some members with dominant group characteristics will respond in ways consistent with *H6–H8*, while we anticipate overall and for marginal group members to respond in ways consistent with *H3–H5*.

### Procedure

We fielded an online survey experiment from June 18 to 28, 2016 comprised of 1,291 respondents. We employed Clear Voice Research to invite an existing online panel of potential participants by email; the response rate was 2.8% (see Supplementary Material). Although this was not a probability-based sample, the sample is more diverse and more representative than alternatives (e.g., Amazon MTurk), and demographically similar to the adult United States population (see Supplementary Material). Our sample was 53.1% female, 57.7% college-educated, 75% white, non-Hispanic, and 37.0% self-identified as Democrats, 36.3% as Independents, and 26.7% as Republicans. The average age was 50.9 years (*SD* = 15.4).

Participants completed the survey experiment via a computer or mobile device. After providing informed consent and affirming that they were 18 years of age or older, the participants completed a brief pre-test questionnaire. The survey pre-test consisted of measures about confidence in the federal government, and the degree to which participants followed certain major news stories. These pre-test measures were asked for a separate study, so we do not use them in the current analyses. The pre-test did not include demographics such as partisanship in order to avoid priming certain identities prior to treatment. After completing the pre-test, all respondents read the following vignette:

“Recently, some local and state governments have debated laws regarding discrimination against various groups, including women, gays and lesbians, African Americans, and transgender people. Gender identity refers to how a person identifies their own gender (as a man, woman, or some other label). For many people, their gender identity may not match their birth sex. For example, a man may identify more as female, or a woman may identify more as male. Transgender is a general term for people whose gender identity or expression is different from their labeled birth sex^1^.”

A previous study showed that similar informational vignettes may reduce discomfort about transgender people and transphobia but had no direct effects on support for transgender rights (Flores et al., 2018). Consistent with previous research (e.g., Miller et al., 2017), we also provided this definition to ensure our respondents had a clear definition of transgender.

#### Recalling Personal Discrimination Exercise

Following the vignette, half of the respondents (*n* = 619) were randomly encouraged to recall a personal experience of discrimination. The other half (*n* = 656) were not prompted and immediately entered the advertisement exposure stage of the experiment. Respondents encouraged to recall discrimination received the following instructions: “Please take a minute to write a few words about a time in your life that you believe you experienced discrimination. What was that like for you?” Two independent examiners rated whether respondents complied with this request (interrater agreement: 79.4%; κ = 0.64) and whether the participants described discrimination based on marginal characteristics or discrimination based on dominant characteristics (interrater agreement: 89.5%; κ = 0.84)^2^. The raters reconciled any disagreements by conferring with each other. Very few respondents failed to comply with this exercise by not writing any response at all, refusing to identify and recall a moment, or writing gibberish (*n*_*compliers*_ = 544; *n*_*non*__–__*compliers*_ = 75). Among those who complied, about half recalled an experience of discrimination based on dominant characteristics (*n* = 263) and half recalled an experience of discrimination based on marginal characteristics (*n* = 281). Importantly, those categorized as recalling an experience based on dominant characteristics primarily raised race (i.e., being white), sex (i.e., being male), or sexuality (i.e., being heterosexual) as the source of their discrimination and frequently attributed blame to affirmative action policies. Those categorized as recalling an experience based on marginal characteristics raised sex (i.e., being female), race or ethnicity (i.e., being Black or Latino), sexuality (i.e., being gay or lesbian), and weight (i.e., being overweight). People spent on average more than 3 min on the exercise, and the average length was 110 characters with those who recalled discrimination based on marginal characteristics writing longer entries than those who wrote based on dominant characteristics. Supplementary Material provide examples of what people wrote.

#### Restroom Advertisements

Following the recall exercise, one-third (*n* = 413) of the respondents were exposed to a campaign advertisement opposing allowing transgender people to access public restrooms consistent with their gender identity. Another third (*n* = 395) of the respondents were exposed to a supportive campaign ad. The remaining third of respondents (*n* = 467) were in a control group that saw no advertisements and directly entered the post-test. The anti-transgender ad came from the 2015 Houston Proposition 1 referendum campaign, which emphasized ominous safety concerns to women and girls if a gender identity inclusive public accommodations ordinance were to be implemented^3^. The ad showed a young girl entering a restroom stall followed by a hooded man, suggesting that such ordinances would allow “any man at any time” an opportunity to harm girls. The pro-transgender ad came from an advertisement aired in response to North Carolina’s HB2 in 2016^4^. This ad showed two cisgender individuals who discuss their eventual acceptance of their transgender male coworker who is seated with them. They elaborated on the discriminatory and unnecessary nature of policies that prohibit transgender people from using restrooms consistent with their current gender identity. The ad also emphasized the revenue loss states face from having such discriminatory policies (i.e., the loss of business North Carolina faced after passing HB2 due to a boycott). Utilizing ads that were deployed in a traditional campaign context increases the external validity of the experiment. However, this comes at the cost of some internal validity because we are unable to pinpoint what exactly about the ads would produce the effects that we observe (e.g., showing a transgender man instead of a transgender woman).

The recall exercise treatment and advertisement treatments were fully factorial among participants producing six distinct groups: a control group (*n* = 239), a recall-only group (*n* = 228), an anti-transgender ad group (*n* = 210), a recall and anti-transgender ad group (*n* = 203), a pro-transgender ad group (*n* = 207), and a recall and pro-transgender ad group (*n* = 188). The groups were balanced by demographics, political attitudes, and religiosity (see Supplementary Table 2).

#### Dependent Variables

Immediately following the advertisements, respondents were asked: “In terms of policies governing public restrooms, do you think these policies should: Require transgender individuals to use the restroom that corresponds with their birth gender OR allow transgender individuals to use the restroom that corresponds with their gender identity?” Overall, 55.1% (*n* = 703) of the respondents preferred the former and 44.9% (*n* = 572) of the respondents preferred the latter. We scored respondents who support transgender individuals using restrooms that correspond with their gender identity as a one, and those who do not as a zero. We chose to limit this dependent variable to restroom access because the ads focused on bathrooms rather a broader range of policies (i.e., gender identity protections in all public accommodations). To overcome the limitation of that dependent variable, the post-test also included twenty additional questions measuring attitudes toward transgender people and rights (see Supplementary Table 3 for question wordings). This included numerous types of non-discrimination protections (e.g., employment, business accommodations, adoption, and general equal protection), and our findings were the same if we analyzed a scale of only these policy questions. We standardized all twenty-one questions, then combined them into a single scale (α = 0.94). We then standardized the scale to have a mean of zero and standard deviation of one with positive scores measuring more favorable attitudes. The scale ranged from −2.4 to 1.9. We analyze the single policy question in addition to the combined scale because the single policy question was most closely related to the advertisements’ content and was the first question asked after administering the treatments. The combined scale ensures that we use all the data in the post-test and that results are not being selectively reported. Even though these data come from 2016 and transgender rights occasionally makes national headlines (e.g., transgender people in the military or in sport), the low salience nature of these topics likely increases the opportunity for people to be open to changing their minds (Tesler, 2015). Indeed, other types of interventions conducted more recently continue to show effects (see Study 2; e.g., Michelson and Harrison, 2020; Kalla and Broockman, 2021).

Following the section measuring attitudes toward transgender people and rights, the post-test also included measures of attitudes toward police, the Zika virus, religion, and respondent demographics. Most of these measures were collected for other studies, and the survey instrument also provided a washout period between treatment and the measurement of respondent demographics.

#### Analysis

We estimated different quantities of interest depending on the treatment group. The Average Treatment Effect (ATE) represents the expected value of the dependent variable for those assigned to a treatment condition compared to the control condition. For the respondents assigned to only view advertisements, the quantity of interest was the ATE. Another quantity of interest was the Intent-to-Treat effect (ITT), which represented the expected value of the dependent variable for those assigned to a treatment condition compared to the control condition. While similar to the ATE, the ITT was estimated when respondents were assigned to a treatment but may not have participated in the treatment (i.e., they did not comply with their assigned treatment). Since some treatment groups were encouraged to recall but some individuals did not, one quantity of interest was the ITT. Another quantity of interest was the Complier Average Causal Effect (CACE), which represented the expected value of the dependent variable for those assigned to a treatment condition and who complied with that treatment compared to those who would have complied in control condition^5^. Since we categorized compliance with the recollection treatment, we also estimated the CACE.

Balance checks suggested that randomization was successful, so we employed contingency table and ordinary least squares (OLS) regression analyses without additional controls on attitudes toward transgender people accessing public restrooms. We also performed OLS regression on the attitudes toward transgender people and their rights scale. For respondents assigned to only view advertisements, this provided estimates of the ATE, and for respondents assigned to recall, this provided an estimate of the ITT. To estimate the CACE in the presence of non-compliance, we estimated two-stage least squares regressions. These estimates contain bootstrapped standard errors estimated from 500 replications. We also report results from two-way ANOVA to test the main and interactive effects of the treatments.

We removed from our analyses any respondent who self-identified as transgender (*n* = 16). We did this because our theoretical motivation is to understand how antidiscrimination exercises influence attitudes toward other marginal out-groups of which a person is not member. Our inferences would have remained the same if we kept transgender respondents in the analysis. We performed our analyses on the remaining sample and separately for members of dominant and marginal groups. Since our theory and hypotheses guided us to consider relative group position, we analyzed white, cisgender, and straight men (dominant group members) separately from women, people of color, and/or LGB people (marginal group members). While there were multiple axes of marginalization (e.g., Cohen, 1999), we considered race, gender, and sexuality to be among the primary characteristics that people use to organize their self-concept, and these categories tended to be protected classes from employment discrimination^6^. Table 1 shows that a majority of dominant-group members recalled a discriminatory experience based on dominant characteristics, while a majority of marginal group members recalled a discriminatory experience based on marginal characteristics. In Supplementary Material, we showed that when considering gender, sexual orientation, race or ethnicity, age, income, partisanship, ideology, and religion, that the only significant predictors of whether discrimination was recalled and the type of discrimination recalled was gender, sexual orientation, and race or ethnicity. Thus, we appropriately categorized the participants to two distinct groups. We did not consider in our causal analyses the qualitative codes of the type of discrimination respondents recalled. Doing so would introduce post-treatment bias (Montgomery et al., 2018) because we only had control over whether participants performed the recall exercise, but we did not have control over how people recalled. There were likely unobservable covariates that would lead people to reflect on dominant characteristics or marginal characteristics that would bias our causal estimates if we included the type of discrimination recalled in our analyses.

**TABLE 1:** The type of discrimination recalled in the exercise (Study 1).

	Women, people of color, or LGB people	White, cisgender, and straight men
Discrimination based on dominant characteristics	34.8% (141)	57.0% (122)
Discrimination based on marginal characteristics	53.1% (215)	30.8% (66)
Did not recall	12.1% (49)	12.2% (26)
*N*	405	214
χ^2^ [2]	34.5**	

Column percentages reported and sample sizes are in the parentheses; degrees of freedom are in brackets. **p < 0.01.

### Results

#### Attitudes on Bathroom Access

A two-way ANOVA on support for transgender bathroom access policies found significant main effects for advertisements [*F*(2,1269) = 24.18, *p* < 0.001], but insignificant main effects for the recall exercise [*F*(1,1269) = 2.47, *p* = 0.12] and their interaction [*F*(2,1,269) = 1.06, *p* = 0.35]. In Table 2, we report the percentage of respondents who support or oppose transgender bathroom access policies by treatment group overall and by dominant and marginal groups. In the control group, a majority opposed transgender people using restrooms consistent with their current gender identity. There were significant differences by treatment condition, with the greatest contrasts from the control among those in the pro-transgender ad conditions and in the recall and anti-transgender ad condition. Table 2 also presents treatment effects. The recall exercise on its own did not increase support for the bathroom issue; respondents in that treatment condition expressed slightly more negative views. The anti-transgender ad on its own had no effect on the issue, but respondents assigned to recall and to view that ad were significantly more opposed to transgender people using restrooms consistent with their gender identity. The effect size was modest (Cohen’s-*d* = −0.29), and about 70 percent of the respondents in that condition were opposed to the issue. There was a 9.5 (90% CI: 1.6, 17.4) percentage point difference in treatment effects between just viewing the anti-transgender ad and viewing the anti-transgender ad with the recall exercise, with attitudes significantly less supportive in the latter condition. The pro-transgender ad conditions significantly increased support for transgender people using restrooms consistent with their current gender identity, and the effects sizes of that ad were similar regardless of whether respondents were assigned to recall (*d* = 0.28) or not (*d* = 0.29). Of all the treatment conditions, the respondents in the pro-transgender ad conditions were the only ones to have a majority favoring transgender people using restrooms consistent with their current gender identity.

**TABLE 2:** Opinions on policies allowing transgender people to use public restrooms based on their current gender identity by treatment group (Study 1).

	Overall					
	Control	Recall exercise	Anti-transgender ad	Recall and anti-transgender ad	Pro-transgender ad	Recall and pro-transgender ad
Support	44.4% (106)	40.4% (92)	49.5% (83)	30.1% (61)	57.9% (120)	58.5% (110)
Oppose	55.2% (133)	59.4% (136)	59.6% (127)	70.0% (142)	42.0% (87)	41.5% (78)
ATE/ITT	–	−0.04 [0.04]^RN,P,RP^	−0.05 [0.04]^RN,P,RP^	−0.14 [0.04]**^,R,N,P,*RP*^	0.14 [0.05]**^,R,N,*RN*^	0.14 [0.05]**^,R,N,*RN*^
CACE	–	−0.05 [0.05]^RN,RP^	–	−0.16 [0.05]**^,R,*RP*^	–	0.16 [0.05]*^,R,*RN*^
*N*	239	228	210	203	207	188
χ^2^ {5}	50.9**					

	**White, Cisgender, and Straight Men**					

Support	39.0% (30)	26.1% (18)	29.6% (21)	25.9% (22)	54.3% (38)	43.3% (26)
Oppose	61.0% (47)	73.9% (51)	70.4% (50)	74.1% (63)	45.7% (32)	56.7% (34)
ATE/ITT	–	−0.13 [0.07]*^,P,*RP*^	−0.09 [0.08]^P,RP^	−0.13 [0.08]*^,P,*RP*^	0.15 [0.08]*^R,N,RN^	0.04 [0.08]^R,N,RN^
CACE	–	−0.15 [0.09]*^,*RP*^	–	−0.15 [0.09]**^,*RP*^	–	0.05 [0.09]^R,RN^
*N*	77	69	71	85	70	60
χ^2^ {5}	19.9**					

	**Women, People of Color, or LGB People**					

Support	46.9% (76)	46.5% (74)	44.6% (62)	33.1% (39)	59.9% (82)	65.6% (84)
Oppose	53.1% (86)	53.5% (85)	55.4% (76)	67.0% (79)	40.2% (55)	34.4% (44)
ATE/ITT	–	−0.004 [0.06]^RN,P,RP^	−0.02 [0.06]^RN,P,RP^	−0.14 [0.06]**^,R,N,P,*RP*^	0.13 [0.06]*^,R,N,*RN*^	0.19 [0.06]**^,R,N,*RN*^
CACE	–	−0.004 [0.06]^RN,RP^	–	−0.15 [0.07]*^,R,*RP*^	–	0.22 [0.07]**^,R,*RN*^
*N*	162	159	139	118	137	128
χ^2^ {5}	34.3**					

Column percentages are reported; sample sizes are in the parentheses; bootstrapped standard errors are in brackets; degrees of freedom are in braces; ATE, Average Treatment Effect; ITT, Intent To Treat effect; CACE, Complier Average Causal Effect. *p ≤ 0.05; **p < 0.01 (one-tailed). ^*R*^, recall; ^*N*^, anti-transgender ad; ^*RN*^, recall and anti-transgender ad; ^*P*^, pro-transgender ad; ^*RP*^, recall and pro-transgender ad. Annotation signifies a significant difference between treatment effects at *p < 0.05; **p < 0.01 (one-tailed).

A two-way ANOVA on support for transgender bathroom access policies among white, cisgender, and straight men found significant main effects for the recall exercise [*F*(1,426) = 4.04, *p* < 0.05] and advertisements [*F*(2,426) = 7.53, *p <* 0.01] but an insignificant interaction [*F*(2,426) = 0.39, *p* = 0.67]. Support for this issue among white, cisgender, and straight men was generally lower compared to support overall with a majority opposing transgender people using restrooms consistent with their current gender identity. There were significant differences by treatment condition, with patterns that differ from the overall results. The recall exercise on its own significantly lowered support for the issue by 13% points. The anti-transgender ad on its own did not differ from the control group. The recall exercise paired with the anti-transgender ad significantly lowered support for the issue by a margin similar to that of solely performing the recall exercise. The pro-transgender ad on its own significantly increased support for transgender restroom access by 15% points, with a majority supporting. The recall exercise paired with the pro-transgender ad, however, did not differ from the control group.

A two-way ANOVA on support for transgender bathroom access policies among women, people of color, or LGB people found significant main effects for the advertisements [*F*(2,837) = 16.1, *p* < 0.01] but an insignificant main effect for the recall exercise [*F*(1,837) = 0.36, *p* = 0.55] and their interaction [*F*(2,837) = 2.08, *p* = 0.13]. Among, women, people of color, or LGB people, support for the issue was similar to overall support with a majority opposing transgender people using restrooms consistent with their current gender identity. There were significant differences by treatment condition. The recall exercise on its own did not significantly affect support for the issue. The anti-transgender ad also did not affect support. The recall exercise paired with the anti-transgender ad, however, significantly lowered support for the issue by 15% points. The treatment effect of the recall exercise paired with the anti-transgender ad was significantly lower than the effect of performing the recall exercise or viewing that ad separately. The pro-transgender ad on its own increased support for the issue by 13% points. The recall exercise paired with the pro-transgender also increased support by 19% points. However, there was not a statistically significant difference between the pro-transgender ad conditions.

#### Attitudes About Transgender People and Transgender Rights Scale

**FIGURE 1:**
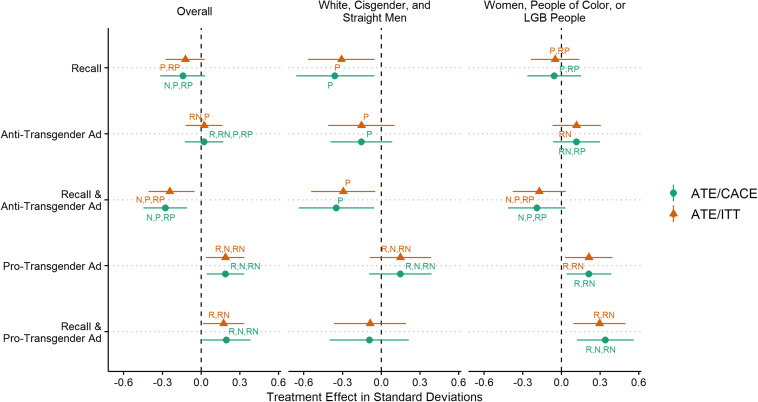
Treatment effects on attitudes toward transgender people and rights scale, Study 1 (ATEs/ITTs and CACEs). R, recall; N, anti-transgender ad; RN, recall and anti-transgender ad; P, pro-transgender ad; RP, recall and pro-transgender ad. Annotation signifies a significant difference in treatment effects at *p* < 0.05 (one-tailed); 90% confidence intervals represented by error bars.

Figure 1 displays treatment effects on the attitudes toward the transgender people and their rights scale. Overall, there was a significant relationship between treatment condition and mean scores on the scale, χ^2^ (5) = 29.06, *p* < 0.01. A two-way ANOVA found significant main effects for the ads [*F*(2,1269) = 10.10, *p* < 0.01] and the recall exercise [*F*(2,1269) = 5.87, *p* = 0.02], but an insignificant interaction [*F*(2,1269) = 1.69, *p* = 0.19]. The recall exercise on its own did not significantly increase scores on the scale; respondents in that treatment condition expressed slightly more negative views. The anti-transgender ad on its own had no significant effect, but respondents assigned to recall and to view the anti-transgender were more negative in their attitudes by 0.25 standard deviations. The difference in treatment effects between just viewing the anti-transgender ad and viewing the ad with the recall exercise was statistically significant. The pro-transgender ad conditions significantly increased scores on the scale by 0.17 standard deviations. The patterns and inferences were consistent when comparing ATEs/ITTs ATEs/CACEs.

Figure 1 also contains treatment effects for dominant and marginal groups. Among white, cisgender, and straight men, there was a significant relationship between treatment condition and mean scores on the scale, χ^2^ (5) = 12.0, *p* < 0.05. A two-way ANOVA on the transgender attitudes scale among white, cisgender, and straight men found significant main effects for the recall exercise [*F*(1,426) = 6.11, *p* = 0.01] and advertisements [*F*(2,426) = 2.64, *p* = 0.07] but an insignificant interaction [*F*(2,426) = 0.3, *p* = 0.74]. Dominant group members assigned to perform the recall exercise were significantly more negative in their attitudes toward transgender people and their rights than the control group by 0.31 standard deviations. Those assigned to only watch the anti-transgender ad were not different from the control. Those assigned to view the anti-transgender ad and perform the recall exercise, however, were significantly more negative in their attitudes than the control by 0.29 standard deviations.

Meanwhile, white, cisgender, and straight men assigned to only view the pro-transgender ad were not different from the control, but the differences in treatment effects were significant between the pro-transgender ad and the recall, anti-transgender ad, or recall and anti-transgender ad conditions. Those assigned to the recall exercise paired with viewing the pro-transgender were not different from the control. Interestingly, there was not a difference among performing the recall exercise and pairing that exercise with either a pro-transgender ad or an anti-transgender ad. The patterns and inferences among dominant group members were consistent when comparing ATEs/ITTs or ATEs/CACEs.

Among women, people of color, or LGB people, there was a significant relationship between treatment condition and mean scores on the scale, χ^2^ (5) = 22.4, *p* < 0.01. A two-way ANOVA on the transgender attitudes scale among women, people of color, or LGB people found significant main effects for the advertisements [*F*(2,837) = 7.39, *p* < 0.01] but an insignificant main effect for the recall exercise [*F*(1,837) = 1.58, *p* = 0.21], and a significant interaction at an α = 0.10 [*F*(2,837) = 2.39, *p* = 0.09]. Marginal group members who were assigned to perform the recall exercise were not different from the control group in their attitudes toward transgender people and rights. Those assigned to view the anti-transgender ad and those assigned to the anti-transgender ad paired with the recall exercise were not different than the control group. There was, however, a significant difference between the treatment effects for the anti-transgender ad condition and the recall with the same ad condition, with attitudes 0.31 standard deviations more negative in the latter group. Those assigned to the pro-transgender ad condition and the recall with the same ad condition had significantly higher scores by 0.21 and 0.29 standard deviations, respectively. The only difference between the ATE/ITT estimates and the ATE/CACE estimates was that those assigned to the recall and pro-transgender ad condition had significantly higher scores than those in the anti-transgender ad condition.

### Discussion to Study 1

Our first two hypotheses examine whether the advertisements persuade our respondents. Interestingly, the ad opposed to transgender rights (one that received wide press coverage for its shock value, see e.g., Oberg, 2015) fails to cause those who view it to be more opposed to transgender rights, so we do not find support for *H1*. This is surprising because public safety concerns tend to be one of the overarching opposition arguments used in mass media to generate opposition to transgender rights (Tadlock, 2014), so people likely should be more receptive to such arguments. Perhaps the reason for no effect is that these arguments are ubiquitous, and the advertisement simply reinforces what people already had accessible in their assessment of this issue (e.g., Zaller, 1992), or that people simply do not find the argument convincing (e.g., Su et al., 2019). However, this ad was used in a campaign, so the proponents of the Proposition 1 referendum campaign must have anticipated the persuasiveness of this argument. Indeed, their campaign won in Houston. Our findings differ from Harrison and Michelson (2017) who found among 443 MTurk workers that safety messages opposed to transgender people accessing bathrooms based on their gender identity significantly lowered support for the issue. A source for this difference may be the differences between the two samples, as MTurk workers tend to be more politically progressive such that pro-transgender arguments may approach a ceiling so greater movement may be available in the negative direction^7^.

Meanwhile, the ad in favor of transgender rights has a positive effect on people’s attitudes, so we find support for *H2*. It is only among those who viewed this ad where there is a majority in support of transgender people using public restrooms consistent with their gender identity. Perhaps challenging the dominant argument by presenting a transgender man, including supportive allies, providing new information, and emphasizing a different set of values alters the considerations relied upon and updates prior beliefs about such policies. The use of a transgender man in the advertisement may be relevant due to a shift in focus to men’s restrooms rather than women’s restrooms (Michelson and Harrison, 2020), given that anti-transgender communications frequently lump men and transgender women into the same predatory category (Westbrook and Schilt, 2014). These mechanisms need to be more extensively unpacked, especially given how consistent these effects are. As we noted, we use these ads to increase the external validity of our findings, but it does come at a cost to internal validity in understanding what components of these ads elicit responses from our participants.

When it comes to recalling personal discrimination, we theorize that the exercise is one that induces relative status threat (Nadler et al., 2009). These exercises are typically designed to increase an individual’s awareness of bias, and thus may help to reduce out-group prejudices (Kalla and Broockman, 2021). Our examples of the type of recollections people wrote indicate the exercise increased identification with broad categories (e.g., white people or women) and threats based on those categories. This is consistent with prior work that indicates that perceiving injustice is linked to social identities (Lalonde and Cameron, 2013). Overall, we do not find an increase in support for transgender people and rights by performing the recall exercise, which does not support *H3*. Contrary to *H4*, the recall exercise may have increased receptivity to the anti-transgender ad as participants in that group have lower support for transgender people and rights than the control. We also do not find the recall exercise to increase receptivity to pro-transgender messages, as the treatment effects are similar to those that did not perform the exercise, which does not support *H5*. The insignificant interaction of the treatments from the ANOVA analyses also do not support *H4* and *H5*. Our results differ from Kalla and Broockman (2021) who show that analogic perspective-taking (i.e., recalling a discriminatory experience with an explicit prompt to link those experiences to out-groups) can reduce prejudices in a survey experiment. This may mean that absent an explicit encouragement to perspective-take respondents maintain a focus on themselves and their identities, do not experience the self-other overlap, and behave in ways consistent with status threat.

Our results suggest that one’s relative group position is important in considering how the recall exercise affects attitudes. We expect that challenging the status of dominant group members would lower attitudes favorable of out-groups, increase the receptivity of anti-transgender messages, and decrease the receptivity of pro-transgender messages because dominant group members respond to such threats in a way to reaffirm the group’s dominant position. We find white, cisgender, and straight men become less accepting of transgender people and rights due to the recall exercise, which supports *H6*. We do not find, however, that the recall exercise paired with the anti-transgender ad induced a stronger negative effect than either the recall exercise or anti-transgender ad on their own, which does not support *H7*. It is noteworthy, however, that only the recall exercise and the recall exercise with the anti-transgender ad results in significantly lowered attitudes than the control. Dominant group members in the pro-transgender ad condition tend to be more supportive of transgender people and rights, but the effect is muted when dominant group members also perform the recall exercise, which supports *H8.* Status threat from the recall exercise consistently lowers attitudes toward the transgender out-group among dominant group members.

We have different expectations for marginal group members. We do not find the recall exercise to increase support among this group, which does not support *H3*. The results of the ANOVA analyses suggest there may be a significant interaction of the recall exercise conditions and ad conditions, which jointly lends some support to *H4* and *H5*, though the substance of this interaction is small. We do not find those who did the exercise and viewed the anti-transgender ad are less persuaded than those who only viewed the anti-transgender ad. Also, we find that the recall exercise paired with the pro-transgender ad increases support for transgender people and rights relative to the control group, but the effect size does not differ from those who only view pro-transgender ad. Thus, the influence of the recall exercise is minimal, and the lack of differences in effect sizes do not individually support *H4* and *H5*.

Our results are understandable considering the psychological reactions people experience when their identities come under threat. People have an individual self-esteem need to cultivate a positive group-based identity and maintain a positive image of that group (Tajfel and Turner, 1979; Brewer, 1991). Part of that maintenance is responding to status threats by finding ways to reposition one’s group in a favorable position. This lends itself to increasing out-group prejudices or, under certain conditions, decreasing them. As debates over transgender rights—especially on public accommodations policies—continue, it remains important to understand the effectiveness of strategies to increase tolerance for marginalized groups including transgender people. The findings from Study 1 imply that some greater care may be necessary when engaging in a broad application of antidiscrimination exercises in order to curtail some of the backfires that may occur (e.g., Stone et al., 2011), and an explicit prompt to perspective-take may be needed (Kalla and Broockman, 2021).

## Study 2: Varying the Perspective-Taking Exercise

Another exercise that has been proposed to reduce prejudice is traditional perspective-taking. In this exercise people imagine their lives as if they were someone who has different and usually marginalized characteristics (Galinsky et al., 2005). The intensity of the perspective-taking exercise has often been quite high. Various exercises have subjects write narrative essays about these thoughts (Galinsky and Moskowitz, 2000; Galinsky et al., 2005; Shih et al., 2009; Gutsell and Inzlicht, 2010; Bruneau and Saxe, 2012), participate in 24 min role-playing games as characters with marginalized characteristics (Simonovits et al., 2018), and hold 10–20 min deep canvassing conversations (Broockman and Kalla, 2016; Kalla and Broockman, 2020, 2021). Recently, Adida et al. (2018) encouraged perspective-taking through a less intensive approach. They had respondents read a vignette about refugees and then they asked respondents to consider what their answers to questions would be if they were refugees. Similarly, Kalla and Broockman (2021) performed an online survey experiment where one condition had respondents perform a traditional perspective-taking exercise with a photo of an out-group member. Both Adida et al. (2018) and Kalla and Broockman (2021) failed to find attitude changes^8^. Thus, it remains a question of whether there exist scalable ways to have people perform a more traditional perspective-taking exercise that results in prejudice reduction.

A common problem in perspective-taking exercises is non-compliance or partial compliance. Non-compliance occurs when participants refuse to do the perspective-taking exercise, and partial compliance occurs when participants engage with the exercise but do not put themselves in the shoes of others. For example, Galinsky and Ku (2004) found 70% of their participants complied by writing narrative essays in first person, but 30% partially complied by writing narrative essays in third person, signaling that they did not imagine their own life as another person. Similarly, after their minimal perspective-taking exercise, Adida et al. (2018) find that a minority in this condition chose to engage in exclusionary behaviors instead of inclusionary ones. People may be more resistant to perspective-take when prompted to take the perspective of stigmatized groups (Todd and Galinsky, 2014). Traditional perspective-taking seems to be contingent on intensity and compliance, and there may be heterogeneous effects of the exercise.

Study 2 extends the scaled-up, traditional perspective-taking exercise by varying the intensity of the exercise in an online survey experiment about transgender people. We also show participants the same advertisements from Study 1, either in favor of or opposed to transgender rights, in order to assess how perspective-taking functions amidst a relevant and contentious political issue^9^. We find that a slightly more intense traditional perspective-taking exercise significantly increases favorable attitudes toward transgender rights. However, on average the effects of perspective-taking are similar to being exposed to favorable ads on their own. Further, our findings suggest that full compliers with the perspective-taking exercise have the strongest positive effects. We then make observational comparisons among full compliers, partial compliers, and non-compliers, which describes who in our study is more resistant to perspective-take.

### Procedure

We fielded an experiment to an adult sample recruited through Dynata (formerly Research Now SSI) during September 9–16, 2019. Dynata invited an existing online panel of potential participants by email. Initially, 3,465 individuals entered into the survey. Early in the survey, we included an attention check for respondents; 1,649 respondents failed this attention check, and they were promptly withdrawn from the survey, resulting in 1,816 initial valid responses. Kung et al. (2018) find that attention checks do not harm the measurement reliability of scales, so the practice of screening participants should increase the internal validity of the experiment (see also Maniaci and Rogge, 2014). Before reaching the embedded experiment an additional 172 respondents exited the survey, resulting in an analytic sample of 1,587 respondents^10^. Our sample was 54.4% female, 40.5% college-educated, 68.2% white, non-Hispanic, 14.9% black, non-Hispanic, 10.4% Hispanic or Latino, 47.0% self-identified as Democrats, and 35.0% self-identified as Republicans. The average age was 46.6 years (*SD* = 16.4).

Participants completed the survey experiment online via a computer or mobile device. After providing informed consent and affirming they were 18 years of age or older, the participants completed a pre-test questionnaire. The pre-test questionnaire consisted of some demographics such as gender, education, race, partisanship, income, voter registration status and other political behaviors, and a variety of attitudinal measures.

#### Perspective-Taking Exercise

After completing the pre-test, respondents were randomly assigned to a condition from a 3 (narrative: perspective-taking vs. modified perspective-taking vs. control) × 2 (advertisement: oppose vs. favor) between participants factorial experimental design with equal probabilities of assignment. One condition was modeled after a traditional perspective-taking (PT) exercise (e.g., Galinsky et al., 2005), with the following prompt:

“Now, imagine that you identify as transgender. Consider the challenges you might face in everyday life. What challenges might you encounter trying to find a good paying job? How do you think your boss and coworkers would treat you? What challenges might you encounter with friends and family members accepting you? What challenges might you encounter in trying to use a public bathroom?”

One-third of the participants (*n* = 552) were provided a space to write a traditional perspective-taking open-ended paragraph style response to these questions. Another condition was a modified perspective-taking (MPT) exercise designed to increase respondent engagement. The one-third of the participants in this condition (*n* = 541) received the same four questions on the same page as the PT exercise. However, instead of a single response space for these questions, each question had its own open-ended space, and participants were required to answer each open-ended question. We expected that the requirement to address each question individually would force participants to engage more intensely with the exercise. The control condition asked the final third of participants (*n* = 551) to respond to the following prompt:

“We’d like to hear a little more about you. Consider a typical day in your life. How is your work situation? How is your family situation? What challenges do you usually encounter?”

The control subjects were given space for an open-ended paragraph style response.

Two independent coders evaluated the write-in responses of the participants. Among those assigned to perspective-take, the coders determined whether they partially complied or fully complied with the treatment. Partial compliance occurred when the response indicated that the participant understood what the prompt was asking of them but refused to perspective-take (e.g., “I can’t imagine what it would like to be transgender” would be coded as partial compliance; whereas, people writing “nsfefkjvk” would be coded as non-compliance). Full compliance with the perspective-taking exercise was coded as those who engaged with the prompt as expected (e.g., they would discuss the difficulties that they would encounter if they were transgender). Between the coders, there was 85.5% agreement with classifying partial compliance (κ =  0.56) and 79.4% agreement with classifying full compliance with perspective-taking (κ =  0.59), and any disagreements were reconciled between the two coders. Among those assigned to perspective-take, 77.6% (*n* = 848) partially or fully complied and 60.8% (*n* = 665) were fully complying perspective-takers.

#### Restroom Advertisements

Following this exercise, all participants were randomized to one of two ad conditions with one ad favorable to transgender rights (*n* = 826) and one ad opposed (*n* = 826). These were the same advertisements used in Study 1. The perspective-taking exercise treatments and advertisement treatments were crossed, producing six distinct groups: a control prompt and anti-transgender ad group (*n* = 275), a control prompt and pro-transgender ad group (*n* = 276), a PT and anti-transgender ad group (*n* = 276), a PT and pro-transgender ad group (*n* = 276), a MPT and anti-transgender ad group (*n* = 267), and a MPT and pro-transgender ad group (*n* = 274).

#### Dependent Variable

After treatment, all participants entered a post-test consisting of a battery of nine attitudinal questions about transgender rights. Question wordings are provided in Supplementary Material. Like Study 1, we combined the standardized questions into a single scale, which was scaled to have a mean of zero and standard deviation of one, with positive values indicating greater levels of support for transgender rights (α = 0.83).

#### Analysis

We estimate four distinct quantities of interest. For those assigned to the control narrative condition and one of the two ads conditions, we estimate the ATE^11^. For those assigned to perspective-take, we estimate the ITT. A difference-in-means estimator is used to estimate these quantities. Among those who partially or fully complied, we also estimate the CACE. These estimates are generated from a two-stage least squares (2SLS) regression, where the perspective-taking exercise is assumed to have no effect on the non-compliers (i.e., the exclusion restriction). Among those who fully complied by perspective-taking, we also estimate the Complier Average Causal Effect for the Effect Class (ECACE), where we expand the exclusion restriction assumption such that there is no effect of perspective-taking on partial compliers (e.g., Sobel and Muthén, 2012). Two-way ANOVAs test the significance of the main and interactive effects of the treatment conditions. Finally, we make observational comparisons in the traits of full compliers, partial compliers, and non-compliers because they are suggestive of who is likely to comply and who may resist or backfire to perspective-taking (e.g., Nyhan and Reifler, 2010). Details of the variables used for traits are provided in the Supplementary Material.

### Results

**FIGURE 2:**
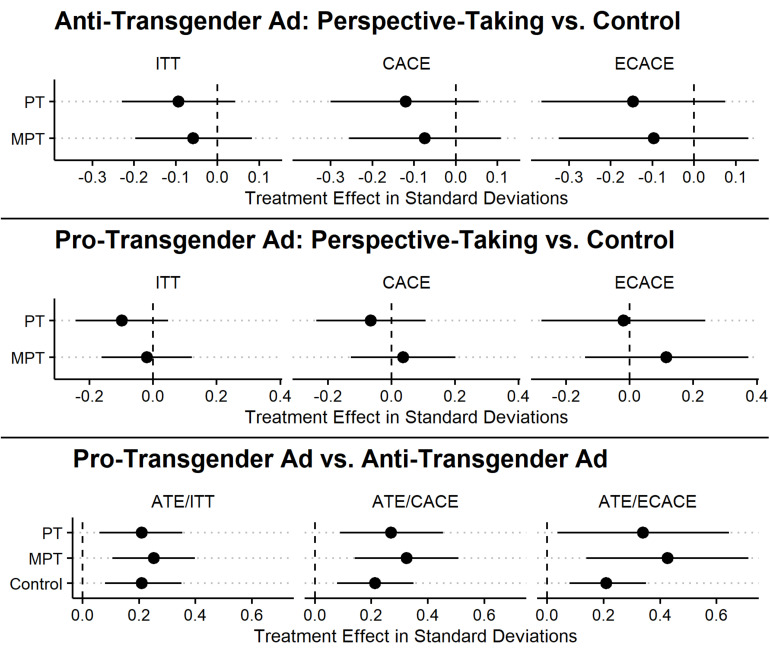
Treatment effect on attitudes toward transgender rights scale, Study 2. 90% confidence intervals represented by error bars.

The ITT analyses are presented in Table 3 with mean scores on the dependent variable and standard deviations and are plotted in Figure 2. While we find that there is a significant relationship between treatment group and attitudes on transgender rights [*F*(5,1581) = 4.56, *p* < 0.01], ANOVA analyses suggest that this was primarily driven by the advertisements [*F*(1,1581) = 20.2, *p* < 0.01] rather than perspective-taking [*F*(2,1581) = 1.25, *p* = 0.29] or their interaction [*F*(2,1581) = 0.07, *p* = 0.93]. This is evident in that those who did not perspective-take had higher mean scores on the outcome than those who did. The PT exercise renders lower mean scores compared to the control or the MPT exercise within each advertisement condition, though these differences are not statistically significant. There tended to be significant differences for those assigned to the pro-transgender ad versus the anti-transgender ad regardless of perspective-taking treatments.

**TABLE 3:** Transgender rights scale, means and standard deviations by treatment group (Study 2).

	Control (*n* = 551)		Traditional perspective-take (PT) (*n* = 552)		Modified perspective-take (MPT) (*n* = 541)	
	*M*	*SD*	*M*	*SD*	*M*	*SD*
Anti-transgender ad (*n* = 818)	−0.06^(a)^	0.93	−0.16^(b)^	0.97	−0.12^(c)^	1.01
Pro-transgender ad (*n* = 826)	0.15^a,b,c^	0.96	0.05^b,c^	1.07	0.13^a,b,c^	1.02

**N* = 1,587; parentheses indicate reference group; letters signify a difference where *p* < 0.05 (one-tailed).*

Table 4 contains regression results from 2SLS regressions with bootstrapped standard errors with 500 replications. The baseline is participants assigned to the control narrative and to view the anti-transgender ad. Model 1 examines treatment effects among those who partially complied or fully complied with the perspective-taking exercises, providing CACE estimates. Most of the CACE estimates are not significantly different from the anti-transgender ad condition, and Figure 2 shows that within ad conditions, PT or MPT are not significantly different from the control. The ATE of the pro-transgender ad indicates that attitudes are more favorable in that condition relative to the anti-transgender ad. The effect of the pro-transgender ad with MPT significantly increased favorable attitudes about transgender rights, though the effect size is not significantly different from the ATE of the pro-transgender ad. We also find that pro-transgender ad conditions tend to be significantly higher than those assigned the view the anti-transgender ad regardless of the perspective-taking assignment. The overall null effects may be due to the combination of partial compliers and full compliers because the former may not have been affected by our perspective-taking exercise.

**TABLE 4:** Regression results on the transgender rights scale (Study 2).

Variable	(1) CACE (Partial + Full)	(2) ECACE (Full)
CT Pro Ad	0.21 (0.09)*^,a,b^	0.21 (0.08)*^,a,b^
PT Anti Ad	−0.12 (0.11)^a,c,d^	−0.15 (0.13)^a,c,d^
PT Pro Ad	0.15 (0.11)^c,e^	0.19 (0.15)^c,e^
MPT Anti Ad	−0.07 (0.12)^b,e,f^	−0.10 (0.15)^b,e,f^
MPT Pro Ad	0.25 (0.11)*^,d,f^	0.33 (0.08)*^,d,f^
Intercept	−0.06 (0.06)	−0.06 (0.06)
*N*	1,587	1,587
Wald-χ^2^ (*df*)	19.9 (5)*	19.7 (5)*
R-squared	0.02	0.03

CT, control; PT, traditional perspective-taking; MPT, modified perspective-taking; bootstrapped standard errors in parentheses; letters signify a difference where *p* < 0.05 (one-tailed). *p < 0.05 (one-tailed).

Model 2 examines treatment effects among those who fully complied, assuming that partial compliers are unaffected similar to non-compliers and providing ECACE estimates. We tend to find patterns that are similar to Model 1. As documented in Figure 2, those assigned to the anti-transgender ad with PT condition and anti-transgender ad with MPT condition remain significantly lower in their attitudes than those in the control, and the ECACE estimates are not statistically significant. Thus, perspective-taking in either form does not weaken the effect of the anti-transgender ad, as we anticipated. While also not significant, the MPT condition relative to the control condition has a slightly larger positive effect among those viewed the pro-transgender ad. Compliers in the pro-transgender with MPT condition have increased support for transgender rights relative to those who viewed the anti-transgender ad, and the ECACE size is slightly larger than the CACE size and almost twice the ITT size.

Since there are differences by varying levels of compliance, we explore the descriptive differences among full compliers, partial compliers, and non-compliers to see if there are characteristics that distinguish these groups by treatment condition. However, these differences are associational and should not be considered causal. Table 5 documents these differences, and all the measures are pre-treatment except for the transgender rights scale, contact measures, race, age, and education. As compared to full compliers in the perspective-taking conditions, partial and non-compliers tend to hold more negative attitudes to transgender rights and have fewer close friends and family members who are gay, lesbian, or transgender. They also tend to have higher scores on racial resentment, hold more traditional beliefs about gender roles, have higher levels of authoritarianism, and identify as men. Some partial compliers also report behaving in more gender conforming ways and are older, while some non-compliers report behaving in gender non-conforming ways and are younger than full compliers. Partial compliers tend to hold more morally traditional values than full compliers. Partial compliers in the traditional PT conditions are also more Republican leaning than full compliers. Thus, many of the traits of individuals who tend to hold negative views of LGB and transgender people (Lewis et al., 2017) relate to whether they tend to resist to perspective-take when prompted.

**TABLE 5:** Comparing the traits of full compliers, partial compliers, and non-compliers (Study 2).

	Anti-transgender ad condition							
	Control comply (*n* = 240)	Control non-comply (*n* = 35)	PT full comply (*n* = 177)	PT partial comply (*n* = 34)	PT non-comply (*n* = 65)	MPT full comply (*n* = 159)	MPT partial comply (*n* = 49)	MPT non-comply (*n* = 59)
**Variable**	***M* (*SD*)**	***M* (*SD*)**	***M* (*SD*)**	***M* (*SD*)**	***M* (*SD*)**	***M* (*SD*)**	***M* (*SD*)**	***M* (*SD*)**

Transgender rights	−0.05(0.93)	−0.16(0.95)	0.02 (1.00)	−0.75(1.02)*	−0.33(0.71)*	0.08 (1.09)	−0.32(0.86)*	−0.48(0.76)*
Gay friend/family	0.60 (0.49)	0.47 (0.51)	0.64 (0.48)	0.50 (0.51)	0.41(0.50)*	0.65 (0.48)	0.50(0.51)*	0.46(0.50)*
Transgender friend/family	0.10 (0.30)	0.26(0.45)*	0.17 (0.38)	0.03(0.18)*	0.17 (0.38)	0.16 (0.37)	0.08 (0.28)	0.16 (0.37)
Racial resentment	0.04 (1.11)	0.00 (0.92)	0.06 (1.24)	0.49(1.25)*	0.10 (0.77)	0.00 (1.17)	0.25 (0.97)	0.12 (0.79)
Non-traditional gender roles	3.97 (1.27)	3.85 (1.38)	4.25 (1.01)	3.49(1.01)*	3.12(1.09)*	4.26 (1.37)	4.02 (1.16)	3.50(1.36)*
Gender non-conformity	0.91 (1.31)	1.54(1.74)*	1.09 (1.35)	0.24(0.43)*	1.63(1.70)*	0.96 (1.45)	0.80 (1.21)	1.15 (1.75)
Disgust	2.83 (0.85)	2.50(0.89)*	2.86 (0.81)	2.70 (0.72)	2.34(0.95)*	2.76 (0.85)	2.73 (0.95)	2.37(0.90)*
Authoritarianism	2.18 (1.34)	2.03 (1.36)	2.09 (1.29)	2.44 (1.31)	2.42(1.12)*	2.12 (1.40)	2.49(1.31)*	2.51(1.15)*
Moral non-traditionalism	−0.03(0.85)	0.25(0.78)*	0.04 (0.89)	−0.46(0.89)*	−0.10(0.56)	0.08 (0.90)	−0.11(0.71)	0.03 (0.60)
Partisanship (Dem. – Rep.)	3.57 (2.08)	3.20 (2.15)	3.73 (2.15)	4.59(2.23)*	3.98 (2.18)	3.53 (2.13)	3.61 (2.08)	3.83 (2.47)
White	0.80 (0.40)	0.73 (0.45)	0.74 (0.44)	0.81 (0.40)	0.59(0.50)*	0.79 (0.41)	0.67 (0.48)	0.70 (0.46)
Female	0.56 (0.50)	0.31(0.47)*	0.62 (0.49)	0.53 (0.51)	0.32(0.47)*	0.63 (0.48)	0.39(0.49)*	0.44(0.50)*
Age	48.6 (16.4)	43.4 (13.9)	46.3 (16.3)	53.5(16.2)*	41.2(14.6)*	48.3 (17.1)	50.2 (14.8)	40.9(14.2)*
College grad	0.43 (0.50)	0.39 (0.50)	0.37 (0.49)	0.35 (0.49)	0.33 (0.48)	0.40 (0.49)	0.51 (0.51)	0.36 (0.48)

	**Pro-transgender ad condition**							
	**Control comply (*n* = 241)**	**Control non-comply (*n* = 35)**	**PT full comply (*n* = 167)**	**PT partial comply (*n* = 49)**	**PT non-comply (*n* = 60)**	**MPT full comply (*n* = 162)**	**MPT partial comply (*n* = 51)**	**MPT non-comply (*n* = 61)**

Transgender rights	0.19 (0.99)	−0.09(0.75)	0.32 (1.07)	−0.41(1.24)*	−0.29(0.65)*	0.40 (1.02)	−0.32(1.00)*	−0.20(0.78)*
Gay friend/family	0.55 (0.50)	0.51 (0.51)	0.59 (0.49)	0.56 (0.50)	0.42(0.50)*	0.62 (0.49)	0.50 (0.51)	0.50 (0.50)
Transgender friend/family	0.17 (0.38)	0.37(0.49)*	0.16 (0.37)	0.10 (0.31)	0.25 (0.44)	0.18 (0.39)	0.02(0.14)*	0.21 (0.41)
Racial resentment	0.00 (1.06)	−0.01(0.80)	−0.10(1.12)	0.49(1.10)*	0.08 (0.92)	−0.18(1.12)	0.37(1.06)*	0.25(0.76)*
Non-traditional gender roles	4.03 (1.40)	3.01(1.48)*	4.20 (1.28)	3.69(1.06)*	3.52(1.37)*	4.39 (1.35)	3.67(1.25)*	3.47(1.23)*
Gender non-conformity	1.00 (1.49)	1.94(2.13)*	1.00 (1.40)	0.49(0.96)*	1.67(2.10)*	1.02 (1.32)	0.84 (1.27)	1.30 (1.66)
Disgust	2.68 (0.91)	2.22(0.94)*	2.74 (0.82)	2.83 (0.84)	2.53 (0.81)	2.76 (0.82)	2.76 (0.94)	2.30(0.92)*
Authoritarianism	2.19 (1.37)	2.23 (1.37)	2.10 (1.35)	2.80(1.17)*	2.58(1.18)*	1.96 (1.38)	2.65(1.31)*	2.05 (1.10)
Moral non-traditionalism	−0.04(0.82)	0.12 (0.84)	0.05 (0.86)	−0.51(0.84)*	−0.04(0.52)	0.10 (0.90)	−0.27(0.80)*	0.09 (0.67)
Partisanship (Dem. – Rep.)	3.87 (2.09)	4.31 (2.34)	3.54 (2.02)	4.29(2.25)*	3.97 (2.32)	3.33 (2.10)	3.71 (2.26)	3.46 (2.26)
White	0.76 (0.43)	0.71 (0.46)	0.80 (0.40)	0.78 (0.42)	0.71 (0.46)	0.76 (0.43)	0.78 (0.42)	0.53(0.50)*
Female	0.59 (0.49)	0.46 (0.51)	0.61 (0.49)	0.49 (0.51)	0.42(0.50)*	0.61 (0.49)	0.41(0.50)*	0.43(0.50)*
Age	46.7 (16.5)	39.6(15.6)*	46.1 (16.9)	52.6(14.6)*	44.5 (17.8)	47.1 (16.8)	45.9 (15.3)	41.3(15.2)*
College grad	0.48 (0.50)	0.46 (0.50)	0.35 (0.48)	0.39 (0.49)	0.31 (0.47)	0.45 (0.50)	0.36 (0.48)	0.33(0.47)*

PT, traditional perspective-taking; MPT, modified perspective-taking; the reference group for significance tests are the full compliers (i.e., Control Comply vs. Control Non-Comply; PT Full Comply vs. PT Partial Comply or PT Non-Comply; MPT Full Comply vs. MPT Partial Comply or MPT Non-Comply). *p < 0.05 (one-tailed).

### Discussion to Study 2

We assessed the effect of political ads on transgender rights. Consistent with *H1* and *H2* we tended to find that attitudes were more negative when people were given the anti-transgender ad and attitudes were more positive when people were given the pro-transgender ad. We further attempted in an online environment to engage people with traditional perspective-taking on what life would be like if they were a transgender person. Our results led us to draw several inferences. First, traditional perspective-taking exercise did little to increase favorable attitudes toward transgender rights. Those assigned to do this exercise and who viewed the anti-transgender ad had lower attitudinal scores relative to those who only viewed the anti-transgender ad, though not statistically significant. Thus, we did not find support for *H4.*

Second, there was more promise in our modified perspective-taking exercise that contained a series of short questions and answers. The modified perspective-taking exercise tended to increase favorable attitudes among those assigned to view the pro-transgender ad, particularly among compliers. We also did not see significantly less supportive attitudes as result of this exercise and viewing the anti-transgender ad. Thus, we find some support for *H4* and *H5* in this context.

However, our results were not dramatic. The effects for perspective-taking were not too different from one another nor were the effects of perspective-taking and viewing the pro-transgender ad much different from only viewing that ad, which does not support *H5*. When separating full compliers from partial compliers, we do find that full compliers generally respond more positively to the pro-transgender ad. These lend some support to *H4* and *H5* among this subpopulation.

Third, our description of partial compliers and non-compliers indicated that there may need to be a consideration of possible negative and heterogeneous effects of perspective-taking and resistance to performing the exercise. Among the respondents who read and understood what they were asked to do but refused to do it, we may have either inadvertently observed a backfire (Nyhan and Reifler, 2010; Wood and Porter, 2019; Merkley, 2020) or treated a subgroup that has characteristics relating to more negative views about transgender people, resulting in their resistance to traditionally perspective-take. Similar to prior research showing resistance to traditional perspective-taking is when the target is from a stigmatized group (Todd and Galinsky, 2014), our observations indicated that the subgroup that held the most negative attitudes toward transgender people were the ones who most resisted reducing their prejudices. This indicates that future research should undertake designs that allow for identification and estimation of causal effects in the presence of varying forms compliance (see e.g., Gerber and Green, 2012).

Perspective-taking is often performed in high-intensity exercises requiring a significant amount of time engaging people and having them build empathic bonds with stigmatized groups. We examined whether more minimal perspective-taking exercises could yield similar effects. We find our modified perspective-taking exercise may have been successful; however, it does not appear to be more effective than only providing pro-transgender ads.

Finally, we draw two broader lessons. First, when attempting to encourage more minimal perspective-taking, researchers should ensure that the exercise is of sufficient intensity by requiring more time and effort. Our MPT exercise only required on average an additional 22 s to complete (see Supplementary Material), but that additional time and effort results in distinct attitudinal responses for those who fully comply. Second, researchers should consider the adverse effects of encouraging perspective-taking and that among some people these attempts may backfire (Wood and Porter, 2019; Merkley, 2020) or be resisted (Todd and Galinsky, 2014).

## General Discussion

The aims of this project were to understand the effects of light touch antidiscrimination exercises on attitudes toward transgender rights and determine whether such exercises moderate people’s receptivity to ads in favor of or opposed to transgender rights. We had respondents reflect on their own experiences of discrimination or traditionally perspective-take. Our findings were mixed regarding their effectiveness. Even though these studies were conducted years apart, we observed patterns common to both.

In study 1, we found political ads in opposition to transgender rights did not result in reduced favorability, inconsistent with *H1*. We also tended to find that political ads in favor of transgender rights changed attitudes to be more favorable, consistent with *H2*. There was a persistent gap in Study 2 between the two ads. If the findings in Study 1 are consistent with Study 2, then the gap is likely more due to the persuasiveness of the pro-transgender ad than the anti-transgender ad^12^. We also found that light touch antidiscrimination exercises at times resulted in distinct attitudinal responses to these advertisements. In theory, this was because the exercises placed people in a distinct cognitive state that made them more or less receptive to the arguments they received from the ads. Our first exercise was to have participants recall their own experiences of discrimination. This exercise did not yield positive attitudinal changes, so it was not supportive of *H3*. We also did not see that those who performed this exercise were less receptive to negative ads or more receptive to positive ads, which was inconsistent with *H4* and *H5*, respectively. Our second exercise was to have participants perspective-take, and we relied a brief intervention and a slightly more intensive intervention. The exercises did not appear to make people less receptive to anti-transgender ads, inconsistent with *H4*. The slightly more intensive intervention led to the largest positive attitude changes when exposed to the favorable ad among those who fully complied with the task, consistent with *H5*. However, our minimal interventions yielded minimal effects overall.

We also observed backfire effects in Study 1 and resistance in Study 2 that deserve greater inquiry. Our first exercise induced white, cisgender, and straight men to be more opposed to transgender rights, more receptive to negative ads, and less receptive to pro-transgender ads, consistent with *H6–H8*. Our second exercise faced resistance among those more likely to hold anti-transgender attitudes.

When it comes to identifying interventions to induce attitude change about stigmatized groups in a political environment with competing messages, attention must be given to what those interventions are and to whom to apply those interventions. Some exercises that may be successful to some in the population may inadvertently backfire with or be resisted by other members of the public. This is important considering that both advocates and academics have pursued broad-scale and generally applicable interventions to reduce out-group prejudices in society, with little regard to differences in sub-populations. Finally, reaching people with political mass communications may, albeit temporarily, be effective in shifting public opinion favorably on transgender rights.

## Data Availability Statement

The raw data supporting the conclusions of this article will be made available by the authors, without undue reservation.

## Ethics Statement

The studies involving human participants were reviewed and approved by the Institutional Review Board – University of Kansas. The patients/participants provided their informed consent to participate in this study.

## Author Contributions

DH-M oversaw data collection. AF performed the statistical analysis and wrote the first draft of the manuscript. All authors contributed to conception and design of the study, manuscript revision, read, and approved the submitted version.

## Conflict of Interest

The authors declare that the research was conducted in the absence of any commercial or financial relationships that could be construed as a potential conflict of interest.

## Publisher’s Note

All claims expressed in this article are solely those of the authors and do not necessarily represent those of their affiliated organizations, or those of the publisher, the editors and the reviewers. Any product that may be evaluated in this article, or claim that may be made by its manufacturer, is not guaranteed or endorsed by the publisher.

## Footnotes

1 We note the language in this informational vignette could be phrased in different ways, and future studies may want to examine how describing transgender people in alternative ways informs and influences the general population.

2 Our independent coders were graduate students of one of the authors.

3 Video available at: https://www.youtube.com/watch?v=D7thOvSvC4E&feature=youtu.be.

4 Video available at: https://www.youtube.com/watch?v=6s3Fx0lq9Ws&feature=youtu.be.

5 For discussion of causal quantities of interest see Gerber and Green (2012).

6 While age and religion are also protected categories, it is unclear whether, for example, older or younger people are marginal. In antidiscrimination law, older individuals are protected (i.e., marginal); however, social constructionists view older generations as advantaged. Similarly, because of variation in which religious groups are dominant in different regions of the country (e.g., LDS dominance in Utah, Southern Baptists and other evangelical Protestants in the South, Roman Catholics in some large cities and the Southwest) or even perhaps less marginalized due to higher localized adherence rates (e.g., Judaism in New York City or Islam in the metro Detroit area), we had no way to determine advantaged versus marginalized religious groups in this data.

7 In their sample, Harrison and Michelson (2017) had 69.3% supporting the issue in the control group compared to 44.4% in our sample.

8 Adida et al. (2018) did find inclusive behavioral changes as a result of the exercise.

9 We further do this because Broockman and Kalla (2016) found that on political measures, perspective-taking on its own did not register a significant change in attitudes. It was only after defining transgender and showing people ads on transgender rights that there were differences between the treated and placebo groups.

10 During treatment, 57 respondents (3%) exited the survey and did not answer the post-test. This attrition was not related to treatment assignment ($\chi_{5}^{2}$ = 1.92,  p = 0.86) and ranged from 8 to 13 respondents per treatment group. Therefore, we use listwise deletion.

11 For a fuller discussion of causal estimates, their assumptions and properties, see Gerber and Green (2012).

12 Further support for this perspective is that the difference in Study 1 between the pro-transgender ad and anti-transgender ad conditions is 0.17 (90% CI: 0.01, 0.32) and in Study 2 it is 0.21 (90% CI 0.08, 0.35).
