# Supplementary material for: Antidiscrimination Interventions, Political Ads on Transgender Rights, and Public Opinion: Results From Two Survey Experiments on Adults in the United States
Source: Front Psychol. 2021 Aug 19;12:729322. doi: 10.3389/fpsyg.2021.729322 (PMC8417058; doi:10.3389/fpsyg.2021.729322)
Supplement: Supplementary file 1 [file Data_Sheet_1.docx]

Supplementary Material

# Advertisement Stimuli

We provide scripts and screenshots from the advertisement stimuli, which we downloaded from YouTube.com (links to the videos provided in the main text). The ad opposed to transgender public accommodations comes from the campaign in Houston, Texas opposing the Houston Equal Rights Ordinance (HERO). A screenshot of the ad is provided in Supplementary Figure 1, and it contained the following text:

“Houston's Proposition One Bathroom Ordinance, what does it mean to you? Any man at any time could enter a woman’s bathroom simply by claiming to be a woman that day. No one is exempt. Even registered sex offenders could follow women or young girls into the bathroom, and if a business tried to stop them, they'd be fined. Protect women’s privacy, prevent danger, vote no on the Proposition One Bathroom Ordinance. It goes too far.”

The ad supportive of transgender public accommodations policies was developed by Freedom for All Americans to emphasize the consequences of North Carolina’s House Bill 2; a bill that repealed a gender identity public accommodations ordinance in Charlotte and also proscribed municipalities from passing gender identity-inclusive accommodations policies. A screenshot of the ad is provided in Supplementary Figure 2, and the ad contained the following text:

“Zeke: All of us take pride in our work, and we're proud to call North Carolina home.

Patricia: I remember when I learned that Zeke was transgender and had transitioned from female to male, and I was a little uncomfortable at first.

Zeke: And I get that.

Chester: I've heard a lot of people are concerned about restrooms.

Patricia: When you stop and think about it everyone needs to use the bathroom just to get through their day, and a law that forces Zeke to use the women's restroom is totally inappropriate and that's exactly what HB 2 does.

Zeke: HB 2 goes so far that if I use the men's room, I could be thrown in jail.

Chester: It’s unnecessary, and it's discriminatory. It took away local protections for gay and transgender people and even protections based on race and gender.

Patricia: HB2 is already hurting our economy. Businesses are leaving and they’re taking jobs.

Zeke: You know conventions have already canceled, and it’s costing us millions. HB 2 just goes against who North Carolinians are and the kind of state we want to live in.”

The first ad was designed to fit in a 30-second advertisement slot; the video actually is 40 seconds in length. The second ad was designed to fit in a 60-second advertisement slot; the video actually is 60 seconds in length.


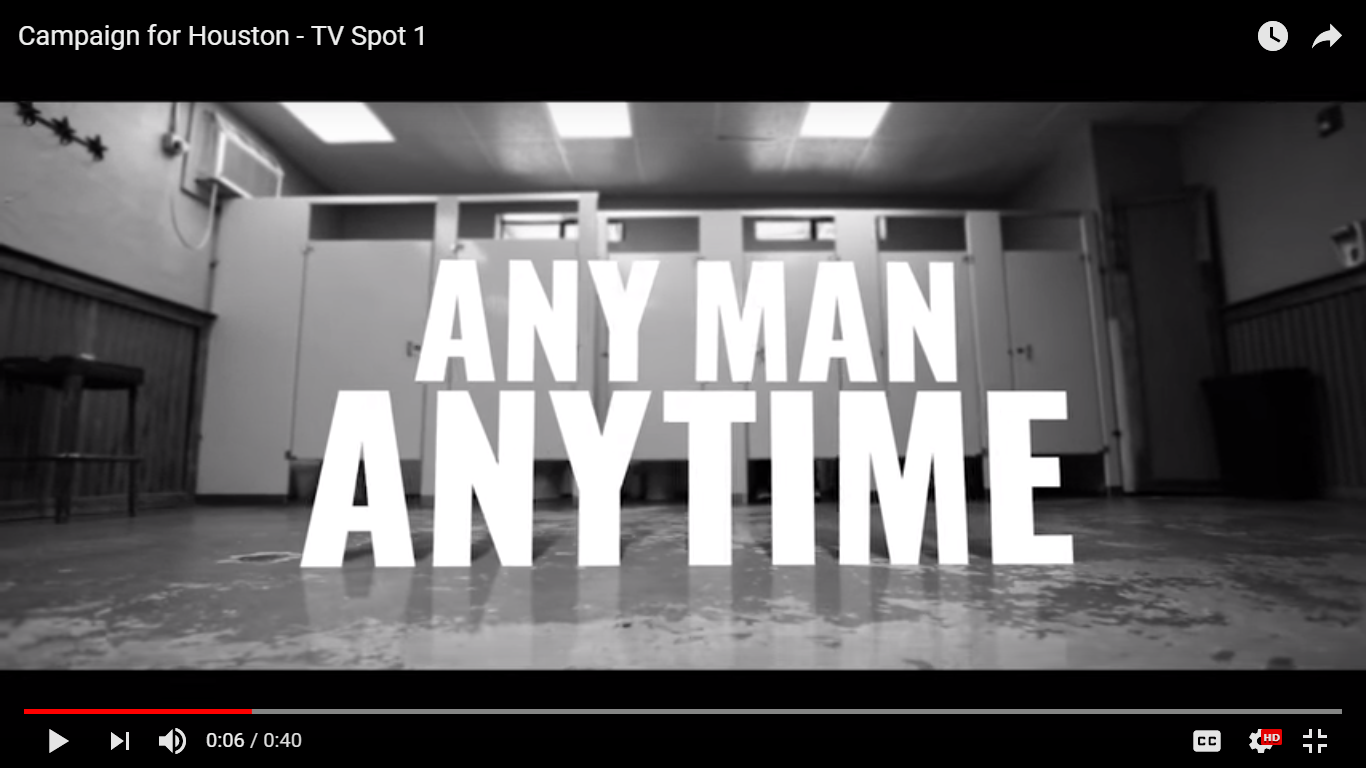


**Supplementary Figure 1.** Screenshot from the advertisement opposed to Gender Identity Inclusive Public Accommodations Protections. Link to video: <https://www.youtube.com/watch?v=D7thOvSvC4E&feature=youtu.be>.


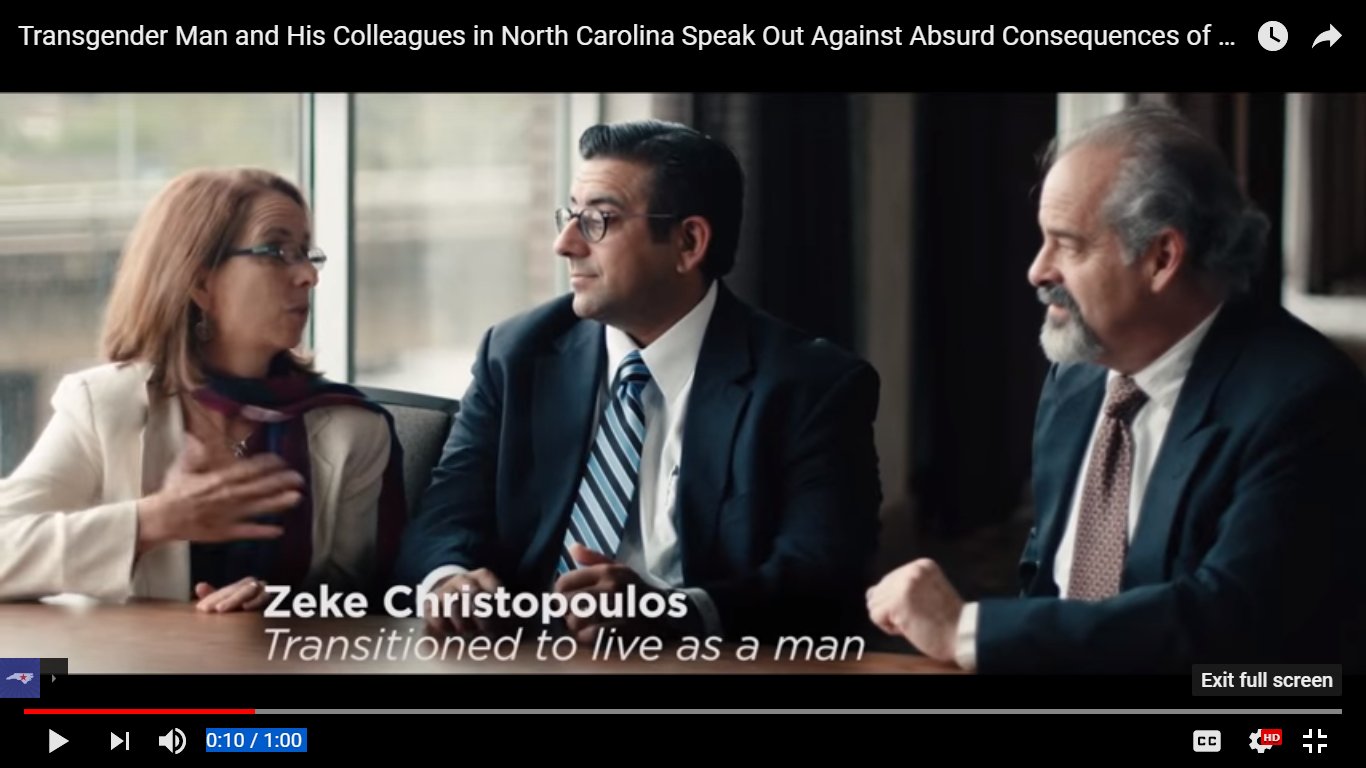


**Supplementary Figure 2.** Screenshot from the advertisement supportive of Gender Identity Inclusive Public Accommodations Protective. Link to the video: <https://www.youtube.com/watch?v=6s3Fx0lq9Ws&feature=youtu.be>.

# Supplementary Details for Study 1

## Further Details on Survey Data Collection for Study 1

Clear Voice Research recruited a sample of 1,290 subjects to participate in a national political study from June 18-28, 2016. Clear Voice has maintained an online panel for the last eight years that is used solely for research purposes. Participants in the panel are told that they will be invited to participate in online research surveys in exchange for various incentives. Their initial registration form collects basic fields including: name, email address, postal address, gender, date of birth, and language. After completing this form, a double opt-in/confirmation email is sent to the email address. Only double opt-in/confirmed accounts are invited to participate in surveys. Following opt-in, panelists are asked to complete their profile so that they collect as many data points as possible, which increases their targeting abilities when they send the member survey invitations. Based on client specifications a sample is pulled in quota group formats. Simple randomization is used to give a representative sample of new and old members within the quota groups. Participants are invited via email to participate in the survey. For this survey, Clear Voice sent out 58,481 invitations, 1,639 began the survey (2.8% response rate) and 1,290 (78.7%) completed the entire survey.

The demographic characteristics of these panels closely resemble that of the United States population on several important traits. Supplementary Table 1 displays the demographics of this sample compared to the American Community Survey 2014 Census estimates, Mechanical Turk samples (adapted from Berinsky et al. 2012) and the Annenberg National Election Study (Johnston et al. 2008). Amazon’s Mechanical Turk is an online marketplace where people hire laborers for a variety of tasks. Since the mid-2000’s researchers have been offering people money to participate in online survey experiments through Amazon’s Mechanical Turk. Recently, scholars have spent considerable effort trying to determine the quality of the samples that are usually obtained through this service (Mullinix et al. 2015). The following table shows that this sample is more representative of the US population on key variables than samples obtained through Amazon’s Mechanical Turk. Moreover, our sample is closer to the U.S. population (American Community Survey 2014) than samples obtained through other means.

Supplementary Table 1. Survey Demographics.

| **Demographics** | **June 2016 Survey** | **ACS 2014 Estimates** | **Mechanical Turk** | **NAES 2008** |
| --- | --- | --- | --- | --- |
| Female | 53.14% | 50.8% | 60.1% | 56.62% |
| Age (mean years) | 50.86 | 37.4 (median) | 20.3 | 50.05 |
| Education (% completing some college) | 57.71% | - | - | 62.86% |
| White | 75.29% | 73.8% | 83.5% | 79.12% |
| Black | 12.39% | 12.6% | 4.4% | 9.67% |
| Asian | 4.26% | 5.0% | - | 2.53% |
| Latino (a) | 5.03% | 16.9% | - | 6.3% |
| Multi-Racial | 1.78% | 2.9% | - | 2.37% |
| Party Identification |  |  |  |  |
| Democrat | 36.95% | - | 40.8% | 36.67% |
| Independent | 36.33% | - | 34.1% | 20.82% |
| Republican | 26.72% | - | 16.9% | 30.61% |
| *N* | 1,291 | - | 484-551 | 19,234 |

## Balance Checks

We report in Supplementary Table 2 *p*-values as it relates to numerous covariates and demographics. Overall, the data appear to be strongly balanced, suggesting successful randomization and completion of the survey.

**Supplementary Table 2. Balance checks.** All *p*-values come from chi-square tests of independence except: ^a^*p*-values from F-test statistics of ordinary least squares regression.

| **Item** | **P-value** |
| --- | --- |
| Religious attendance | 0.496 |
| Religious importance | 0.294 |
| Religious identification | 0.984 |
| Party Identification (3 categories) | 0.550 |
| Party Identification (7 categories) | 0.537 |
| Ideology | 0.293 |
| Age Group | 0.517 |
| Female | 0.147 |
| Race or Ethnicity | 0.335 |
| Income | 0.309 |
| 2016 Presidential Vote Choice (intention) | 0.710 |
| Interest in Politics | 0.703 |
| Children at home | 0.420 |
| Identify as transgender | 0.457 |
| Sexual orientation | 0.225 |
| Political knowledge^a^ | 0.442 |
| Age (continuous)^a^ | 0.666 |
| Income (continuous)^a^ | 0.933 |

## Question Wordings for the Dependent Variables

After administering treatment, the post-test contained 21 items measuring attitudes toward transgender people and transgender rights. Supplementary Table 3 contains the question wordings for each item. An exploratory factor analysis resulted in two eigenvalues greater than one. All the items had decent factor loadings on the first factor except for Q10_2_6. Upon evaluation of the second factor, it was not apparent that any set of items distinctively loaded onto that second factor and the largest factor loading was 0.38. Therefore, we decided to create an additive scale of all 21 items (α=0.93), reverse coding the items as appropriate such that larger values corresponded with supportive attitudes about transgender people and rights. The scale was rescaled to have a mean of zero and standard deviation of one. Supplementary Figure 3 contains density plots of the transgender attitudes scale by treatment group for the full sample and subdivided by dominant and marginal groups members.

**Supplementary Table 3. Question wordings.**

| Variable | Wording | Response Set |
| --- | --- | --- |
| Q10_1 | In terms of policies governing public restroom, do you think these policies should: | - Require transgender people to use the restroom that corresponds with their birth gender  - Allow transgender individuals to use the restroom that corresponds with their gender identity |
| Q10_2 | How much do you agree or disagree with the following statements: |  |
| Q10_2_1 | Allowing transgender individuals to use the restroom of their choice poses a security risk for women and children. | Strongly Agree – Strongly Disagree (7 pts.) |
| Q10_2_2 | It discriminates against transgender individuals to require them to use the restrooms that correspond to their birth gender. | Strongly Agree – Strongly Disagree (7 pts.) |
| Q10_2_3 | The idea of using a restroom with a transgender person disgusts me. | Strongly Agree – Strongly Disagree (7 pts.) |
| Q10_2_4 | The safety of transgender individuals is put at risk if they are required to use restrooms that correspond with their birth gender. | Strongly Agree – Strongly Disagree (7 pts.) |
| Q10_2_5 | It’s simple: men should use the men’s restroom and women should use the women’s restroom. | Strongly Agree – Strongly Disagree (7 pts.) |
| Q10_2_6 | It makes me uncomfortable to use a restroom in public no matter who else is using it. | Strongly Agree – Strongly Disagree (7 pts.) |
| Q10_3 | Are you, personally, comfortable or uncomfortable when you are around someone who is transgender? | - Comfortable  - Somewhat comfortable  - Somewhat uncomfortable  - Uncomfortable |
| Q10_4 | Below are a few statements about transgender people. Please tell us how much you agree or disagree with each one. |  |
| Q10_4_1 | Transgender people should be allowed to change the sex listed on their Driver's License or state ID card. | - Completely agree  - Agree  - Disagree  - Completely disagree |
| Q10_4_2 | Congress should pass laws to protect transgender people from job discrimination. | - Completely agree  - Agree  - Disagree  - Completely disagree |
| Q10_4_3 | Schools should NOT allow transgender students to use restrooms that match with their gender identity. | - Completely agree  - Agree  - Disagree  - Completely disagree |
| Q10_4_4 | Insurance companies should NOT be required to pay for medical treatments related to transgender health issues. | - Completely agree  - Agree  - Disagree  - Completely disagree |
| Q10_4_5 | Laws should protect transgender children from bullying in schools. | - Completely agree  - Agree  - Disagree  - Completely disagree |
| Q10_4_6 | Businesses should have the right to refuse services to transgender people based on religious beliefs. | - Completely agree  - Agree  - Disagree  - Completely disagree |
| Q10_4_7 | Transgender persons deserve the same rights and protections as other Americans. | - Completely agree  - Agree  - Disagree  - Completely disagree |
| Q10_4_8 | Transgender people should be allowed to serve openly in the military. | - Completely agree  - Agree  - Disagree  - Completely disagree |
| Q10_4_9 | Parents should let their child express a transgender identity. | - Completely agree  - Agree  - Disagree  - Completely disagree |
| Q10_4_10 | Students who have had a sex change should NOT be allowed to play college sports. | - Completely agree  - Agree  - Disagree  - Completely disagree |
| Q10_4_11 | A local business should have a right to NOT hire a transgender person. | - Completely agree  - Agree  - Disagree  - Completely disagree |
| Q10_4_12 | Parents should assist their child in expressing a transgender identity by allowing doctors to give the child hormone blockers to delay puberty. | - Completely agree  - Agree  - Disagree  - Completely disagree |
| Q10_4_13 | Transgender people should NOT be allowed to adopt children. | - Completely agree  - Agree  - Disagree  - Completely disagree |

**
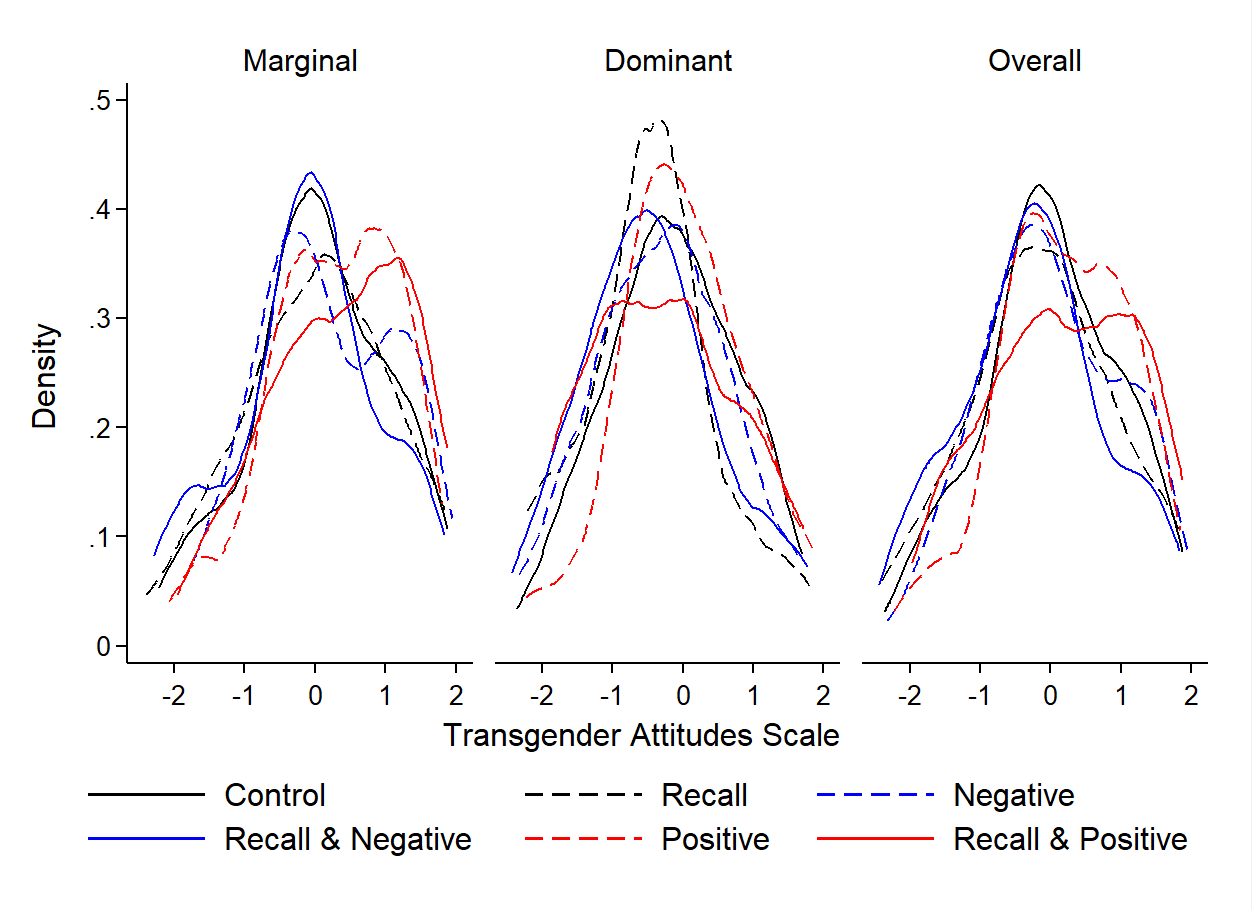
**

**Supplementary Figure 3. Density plots of the transgender attitudes scale by treatment group.**

## Multinomial Logistic Model Predicting the Type of Discrimination Recalled

In the main text, we compared how white, cisgender-straight men recalled an experience of discrimination as either one that was based on dominant or marginal characteristics to that of women, people of color, and LGB people. Supplementary Table 4 reports regression results from a multinomial logistic regression predicting the type of discrimination recalled with the baseline being that respondents recalled discrimination based on marginal characteristics. We find that women were more likely to recall discrimination than others, and Asian Americans were more likely to not recall discrimination. Other demographics, including ideological self-placement and partisan identification, do not predict whether respondents did not recall an experience of discrimination. Interestingly, relative to expressing discrimination based on marginal characteristics, women, black respondents, LGB people, and those with greater educational attainment were less likely to express discrimination based on dominant characteristics. Partisanship and ideology also have little relationship to whether people expressed discrimination based on dominant characteristics. People from historically marginalized groups or with greater educational attainment were more likely to express discrimination based on marginal characteristics.

**Supplementary Table 4: Multinomial logistic regression predicting non-compliance and expressions of discrimination based on dominant characteristics relative to expressions of discrimination based on marginal characteristics.** Standard errors are in parentheses; ^*p*<0.10; **p*<0.05; ***p*<0.01; ****p*<0.001 (two-tailed).

| Variables | Did not recall | Discrimination based on dominant characteristics |
| --- | --- | --- |
| Age | -0.009 (0.0097) | 0.008 (0.007) |
| Female | -0.78 (0.27)** | -0.82 (0.18)*** |
| Black | -0.15 (0.42) | -1.35 (0.38)*** |
| Asian | 1.18 (0.56)* | -0.27 (0.52) |
| Native American | -0.35 (1.12) | -1.92 (1.10)^ |
| Hispanic or Latino | 0.62 (0.51) | -0.19 (0.42) |
| Multiracial | 0.29 (1.15) | 0.20 (0.70) |
| H.S. Graduate | -1.07 (1.10) | -1.37 (0.88) |
| Some College | -1.11 (1.06) | -1.94 (0.87)* |
| College Graduate | -1.14 (1.09 | -1.72 (0.88)^ |
| Graduate/Professional School | -1.02 (1.12) | -1.85 (0.91)* |
| Income | -0.008 (0.10) | 0.01 (0.07) |
| Ideology (Liberal to Conservative) | -0.07 (0.11) | 0.10 (0.08) |
| Partisanship (Democrat to Republican) | 0.007 (0.09) | 0.008 (0.06) |
| Does Not Have Kids | -0.21 (0.31) | 0.05 (0.23) |
| LGB | -0.69 (0.66) | -1.01 (0.48)* |
| Christian | -0.05 (0.29) | -0.10 (0.20) |
| Intercept | 1.00 (1.23) | 2.11 (1.41) |
| *N* | 619 | |
| Likelihood Chi-square | 84.2, 34 degrees of freedom | |
| Pseudo-R-squared | 0.07 | |

## Regression Results in Table Form

We report in Supplementary Table 5 regression results on opinions on whether transgender people should be allowed to use restrooms consistent with their gender identity, which represents ATEs and ITTs. We also present results in Supplementary Table 6 of the ATEs and CACEs from two-staged least squares regression. Supplementary Table 7 and Supplementary Table 8 report the regression results for the attitudes toward transgender people and rights scale.

**Supplemental Table 5. Regression results on support for transgender people to use restrooms consistent with their gender identity (ATE/ITT).** OLS regression; estimates are ATEs and ITTs; bootstrapped standard errors are in the parentheses; *p<0.05; **p<0.01 (one-tailed).

| Variable | (1)  Overall | (2)  White, Cisgender, and Straight Men | (3)  Women, People of Color, LGB People |
| --- | --- | --- | --- |
| Recall | -0.04 (0.04) | -0.13 (0.08)* | -0.004 (0.06) |
| Negative Ad | -0.05 (0.04) | -0.09 (0.08) | -0.02 (0.06) |
| Recall with Negative Ad | -0.14 (0.04)** | -0.13 (0.07)* | -0.14 (0.06)* |
| Positive Ad | 0.14 (0.05)** | 0.15 (0.08)* | 0.13 (0.06)* |
| Recall with Positive Ad | 0.14 (0.05)** | 0.04 (0.09) | 0.19 (0.06)** |
| Intercept | 0.44 (0.03) | 0.39 (0.05) | 0.47 (0.04) |
| *N* | 1,275 | 432 | 843 |
| $R^{2}$ | 0.04 | 0.05 | 0.04 |
| $\chi_{5}^{2}$ | 55.3** | 18.32** | 38.24** |

**Supplementary Table 6. Regression results on support for transgender people to use restrooms consistent with their gender identity (ATE/CACE).** Two-stage least squares regression; estimates are ATEs and CACEs; bootstrapped standard errors are in the parentheses; *p≤0.05; **p<0.01 (one-tailed).

| Variable | (1)  Overall | (2)  White, Cisgender, and Straight Men | (3)  Women, People of Color, LGB People |
| --- | --- | --- | --- |
| Recall compliers | -0.05 (0.05) | -0.15 (0.09)* | -0.004 (0.06) |
| Negative Ad | -0.05 (0.04) | -0.09 (0.08) | -0.02 (0.06) |
| Recall compliers with Negative Ad | -0.16 (0.05)** | -0.15 (0.09)* | -0.15 (0.07)* |
| Positive Ad | 0.14 (0.05)** | 0.15 (0.08)* | 0.13 (0.06)* |
| Recall compliers with Positive Ad | 0.16 (0.06)** | 0.05 (0.09) | 0.22 (0.07)** |
| Intercept | 0.44 (0.03) | 0.39 (0.06) | 0.47 (0.04) |
| Stage one regression (DV = compliance) |  |  |  |
| Recall | 0.87 (0.01)** | 0.87 (0.02)** | 0.87 (0.02)** |
| Recall with Negative Ad | 0.88 (0.01)** | 0.85 (0.03)** | 0.90 (0.01)** |
| Recall with Positive Ad | 0.89 (0.02)** | 0.93 (0.02)** | 0.87 (0.02)** |
| *N* | 1,275 | 432 | 843 |
| $R^{2}$ | 0.04 | 0.05 | 0.03 |
| $\chi_{5}^{2}$ | 50.64** | 23.18** | 35.27** |

**Supplementary Table 7. Regression results on supportive attitudes for transgender people and rights(ATE/ITT).** OLS regression; estimates are ATEs and ITTs; bootstrapped standard errors are in the parentheses; *p<0.05; **p<0.01 (one-tailed).

| Variable | (1)  Overall | (2)  White, Cisgender, and Straight Men | (3)  Women, People of Color, LGB People |
| --- | --- | --- | --- |
| Recall | -0.12 (0.09) | -0.31 (0.16)* | -0.04 (0.11) |
| Negative Ad | 0.02 (0.09) | -0.15 (0.16) | 0.12 (0.11) |
| Recall with Negative Ad | -0.24 (0.10)** | -0.29 (0.15)* | -0.17 (0.13) |
| Positive Ad | 0.19 (0.09)* | 0.15 (0.14) | 0.21 (0.11)* |
| Recall with Positive Ad | 0.17 (0.10)* | -0.09 (0.17) | 0.30 (0.12)** |
| Intercept | -0.01 (0.06) | -0.15 (0.11) | 0.06 (0.08) |
| *N* | 1,275 | 432 | 843 |
| $R^{2}$ | 0.02 | 0.03 | 0.02 |
| $\chi_{5}^{2}$ | 29.06** | 12.04* | 22.42** |

**Supplementary Table 8. Regression results on supportive attitudes for transgender people and rights (ATE/CACE).** Two-stage least squares regression; estimates are ATEs and CACEs; bootstrapped standard errors are in the parentheses; *p≤0.05; **p<0.01 (one-tailed).

| Variable | (1)  Overall | (2)  White, Cisgender, and Straight Men | (3)  Women, People of Color, LGB People |
| --- | --- | --- | --- |
| Recall compliers | -0.14 (0.11) | -0.36 (0.18)* | -0.06 (0.13) |
| Negative Ad | 0.02 (0.09) | -0.15 (0.14) | 0.12 (0.11) |
| Recall compliers with Negative Ad | -0.28 (0.10)** | -0.35 (0.18)* | -0.19 (0.14) |
| Positive Ad | 0.19 (0.06)* | 0.15 (0.15) | 0.21 (0.08)* |
| Recall compliers with Positive Ad | 0.20 (0.11)* | -0.09 (0.15) | 0.34 (0.13)** |
| Intercept | -0.01 (0.06) | -0.15 (0.10) | 0.06 (0.08) |
| Stage one regression (DV = compliance) |  |  |  |
| Recall | 0.87 (0.01)** | 0.87 (0.02)** | 0.87 (0.02)** |
| Recall with Negative Ad | 0.88 (0.01)** | 0.85 (0.03)** | 0.90 (0.01)** |
| Recall with Positive Ad | 0.89 (0.02)** | 0.93 (0.02)** | 0.87 (0.02)** |
| *N* | 1,275 | 432 | 843 |
| $R^{2}$ | 0.02 | 0.03 | 0.02 |
| $\chi_{5}^{2}$ | 27.18** | 13.61* | 19.64** |

## Engagement with the Recall Exercise

Half of the participants (n = 628) were assigned to perform a recall exercise of an experience in which they felt they were discriminated. We report summary measures of their engagement with this prompt in Supplementary Table 9 by assessing the length of time they spent and also the number of characters they used in responding to the prompt. Since these measures have a high skew, we report results from median regressions in addition to traditional OLS regressions. The average length of time was over three minutes, and the median length of time was over one minute. There were not statistically significant differences by type of reflection or if the respondents were white, cisgender, and straight men or not. There were significant differences in the length of entry. The average length of entry was about 110 characters, and the median length was about 90 characters, which is about one to two sentences. People of color, LGBT people, or women who recalled discrimination based on marginal characteristics wrote a significantly longer entry than recalling an experience based on dominant characteristics or white, cisgender, and straight men. Thus, there were not too many differences among participants in their engagement with the exercise, except for the recall exercise among marginal group members. Supplementary Table 10 also reports regression results by demographics and type of discrimination recalled. We find that Black and Native Americans had higher medians in their length of time on the exercise than others. We also find that entries are longer if participants recalled an experience based on marginal characteristics. Women also tend to write longer entries than men by about 20 characters. Asian American wrote less in their entry.

Supplementary Table 11 provides five additional entries of each qualitative code (i.e., noncompliance, discrimination based on dominant characteristics, and discrimination based on marginal characteristics). Noncompliance is reflected with no information or engagement with the prompt. Discrimination based on dominant characteristics cite being straight, male, and white as the characteristics that caused the discrimination. Discrimination based on marginal characteristics cite being a woman, person of color, overweight, or LGB as a source of the discrimination. As we explain in the main text, it is clear that the exercise primed certain identities and characteristics, which has the potential to produce the variety of emotional and other cognitive responses that have been documented when people perceive injustice.

**Supplementary Table 9. Regression results on length of time and entry to the recall exercise.** WCSM = White, Cisgender, and Straight Men; ^*p* < .10; **p* < .05; ***p* < .01; ****p* < .001.

|  | Length of time (seconds) | | | | Length of entry (characters) | | | |
| --- | --- | --- | --- | --- | --- | --- | --- | --- |
| Variables | (1) | (2) | (3) | (4) | (5) | (6) | (7) | (8) |
|  | OLS | OLS | Median | Median | OLS | OLS | Median | Median |
| *Type of discrim.* |  |  |  |  |  |  |  |  |
| Dominant | 0.03 (103.8) | 20.7 (131.2) | -9.4  (9.3) | -15.0 (12.9) | -15.7 (9.9) | -22.4 (12.4)^ | -23.0 (15.0) | -20.0 (18.6) |
| Marginal | -41.8 (103.0) | -68.0 (125.2) | -1.9  (9.2) | -7.1  (12.3) | 43.8*** (9.8) | 47.5*** (11.9) | 57.0*** (14.9) | 73.0*** (17.8) |
| WCSM | -78.6 (68.6) | -97.9 (192.0) | -1.25  (6.1) | -10.7 (18.8) | -8.9 (9.8) | -10.1 (18.2) | -13.0 (9.9) | 6.0 (27.3) |
| Dominant * WCSM | -- | -39.7 (215.4) | -- | 11.5 (21.1) | -- | 14.8 (20.4) | -- | -7.0 (30.6) |
| Marginal * WCSM | -- | 102.1 (221.9) | -- | 11.9 (21.8) | -- | -16.1 (21.1) | -- | -48.0 (31.5) |
| Intercept | 209.4 (94.3)* | 216.1 (113.0)^ | 85.7*** (8.4) | 90.4*** (11.1) | 109.8*** (9.0) | 110.2*** (10.7) | 91.0*** (13.6) | 83.0*** (16.1) |
| *N* | 619 | 619 | 619 | 619 | 619 | 619 | 619 | 619 |
| *F*  (*df*, *df*) | 0.50  (3, 615) | 0.48  (5, 613) | -- | -- | 31.8*** (3, 615) | 20.13*** (5, 613) | -- | -- |
| Psuedo-*R*^2^ | 0.002 | 0.004 | 0.002 | 0.003 | 0.13 | 0.14 | 0.10 | 0.11 |

**Supplementary Table 10. Regression results on length of time and entry to the recall exercise, by demographics.** ^*p* < .10; **p* < .05; ***p* < .01; ****p* < .001.

|  | Length of time (seconds) | | Length of entry (characters) | |
| --- | --- | --- | --- | --- |
| Variables | (1) | (2) | (3) | (4) |
|  | OLS | Median | OLS | Median |
| Dominant | 71.5 (104.7) | -2.2 (9.8) | -16.8 (9.9)^ | -16.6 (15.8) |
| Marginal | 9.5 (103.3) | 2.1 (9.7) | 39.9 (9.8)*** | 56.5 (15.5)*** |
| Age | -4.1 (2.3) | 0.2 (0.2) | 0.1 (0.2) | -0.1 (0.3) |
| Female | 55.1 (65.1) | 4.8 (6.1) | 19.2 (6.2)** | 24.3 (9.8)* |
| LGBT | -6.5 (152.7) | -8.7 (14.3) | -12.8 (14.4) | 1.8 (23.0) |
| Black | 95.6 (112.1) | 42.9 (10.5)*** | 10.0 (10.6) | 14.2 (16.9) |
| Asian American | 782.4 (169.5)*** | 5.0 (15.9) | -45.4 (16.0)** | -43.9 (25.5)^ |
| Latino | -3.9 (140.8) | -1.8 (13.2) | -6.9 (13.3) | -19.8 (21.2) |
| Native American | 66.9 (280.9) | 71.3 (26.3)*** | 11.0 (26.6) | 40.2 (42.3) |
| Multiracial | 1.9 (251.8) | 15.4 (23.6) | -21.8 (23.8) | -1.1 (37.9) |
| HS Grad | 1.8 (265.1) | -39.5 (24.8) | -41.6 (25.1)^ | -58.0 (39.9) |
| Some College | 29.2 (260.4) | -30.8 (24.4) | -23.1 (24.6) | -40.3 (39.2) |
| Bachelor’s | 86.4 (265.5) | -40.7 (24.8) | -18.8 (25.1) | -36.5 (40.0) |
| Post-graduate | -53.9 (274.3) | -30.4 (25.7) | -0.8 (26.0) | -4.5 (41.3) |
| Income | 4.2 (25.2) | 0.8 (2.4) | 3.5 (2.4) | 2.8 (3.8) |
| Ideology | -19.4 (26.3) | -0.11 (2.5) | 0.1 (2.5) | 3.5 (4.0) |
| Partisanship | 8.8 (21.6) | -1.4 (2.0) | -0.1 (2.0) | -2.6 (3.3) |
| No Kids | 85.2 (79.0) | -0.7 (7.4) | -0.4 (7.5) | 4.1 (11.9) |
| Intercept | 216.1 (308.2) | 103.5 (28.8)*** | 108.0 (29.2)*** | 99.3 (46.4)* |
| *N* | 619 | 619 | 619 | 619 |
| *F* (*df*, *df*) | 1.80 (18,600)* | -- | 7.66 (18,600)*** | -- |
| Pseudo-*R*^2^ | 0.05 | 0.02 | 0.19 | 0.13 |

**Supplementary Table 11. Additional examples of entries coded by noncompliance or discrimination based on dominant or marginal characteristics.**

| Type | Entry |
| --- | --- |
| Noncompliance | “No comments” |
| Noncompliance | “76rt7809-oihgf75r76t87y9809jhgoufiyudtryytuop[lkpjohiguof7rt689=-oiuytr767t8yu” |
| Noncompliance | “hikjhnikljnikljolkjmolk,jml” |
| Noncompliance | “fdgetgfgfdg ndsandsabdb bdsbfjaje jsdbfbds jksfnmnmasd dsajfbdbfnsa fkdfjdshfsdfbnbfr dsnfbbdsfbfrfsgfgfdgfgf sdfdsfsrfegsdf ffwefdfdf fregfgfkjsakdjasdh jhasdjsbrghsdg kjasdjasjkldjklaljks ljkalsjkdjk jkalsdjkl jklfjklsdjkf jlksdfjkl kjljklasj” |
| Noncompliance | “N/A” |
| Dominant | “I've experienced discrimination from a gay manager before. Because I wasn't "one of the girls" (I'm a man), he tried to get rid of me, even though he could give his manager no reason to legally fire me. He just wanted rid of me because I wasn’t gay.” |
| Dominant | “As a white male, I don't think I have ever experienced discrimination. I was raised in a slum area, and perhaps, some thought less of me because I came from such an area” |
| Dominant | “i applied for a job but did not get it because i was white and a male” |
| Dominant | “When trying to apply to become a LA county fireman. Because of affirmative action I was told that I could ace the exam is still not be considered for a position because I was white.” |
| Dominant | “I applied for the same summer job I had the year before. I had lots of experience and would have done better than the year before. I was denied the job because I am white and they needed to hire more minority students. I felt I was robbed.” |
| Marginal | “I wanted to play guys football and it took a meeting and a special try out for me to play considering im a girl and its a boys team the result was i played” |
| Marginal | “as a lesbian and as a womna, discrimation, overt and covert, can pop up at any time - this is very frustrating and annoying - does not feel good = feels goof when can res[omded with a sense of em[powerment” |
| Marginal | “I've always been overweight. Since the 2nd grade and now I am 63. A lot of prejudice and discrimination doesn't even consider obesity as a part of discrimination. Believe me it is. People look at me like I am a glutton if we are out in public” |
| Marginal | “I am Hispanic and experienced a fair amount of discrimination in my life.” |
| Marginal | “when I was not allowed in an all whit neighborhood as a kid. It made me feel sad that I was not invited or appreciated” (Note: respondent identified as Black) |

# Supplementary Details for Study 2

## Question Wordings

The question wordings of the dependent variables are provided in Supplementary Table 12. Supplementary Table 13 contains question wordings for the traits documented in Table 5. For any additive scales in Supplementary Table 13, we also report reliability statistics. We provide summary statistics of these variables in Supplementary Table 14.

**Supplementary Table 12. Question wordings.**

| Variable | Wording | Response Set |
| --- | --- | --- |
| Q18_3_1 | Laws should protect transgender children from bullying in schools. | 1 – Completely agree; 2 – Agree  3 – Disagree; 4 – Agree |
| Q18_3_2 | Businesses should have the right to refuse services to transgender people based on religious beliefs. | 1 – Completely agree; 2 – Agree  3 – Disagree; 4 – Agree |
| Q18_3_3 | Transgender people deserve the same rights and protections as other Americans. | 1 – Completely agree 2 – Agree  3 – Disagree; 4 – Agree |
| Q18_3_4 | Transgender people should only be allowed to use public restrooms that are consistent with the sex listed on their driver’s license/state ID card. | 1 – Completely agree; 2 – Agree  3 – Disagree; 4 – Agree |
| Q18_3_5 | Transgender people should be allowed to serve openly in the military. | 1 – Completely agree; 2 – Agree  3 – Disagree; 4 – Agree |
| Q18_3_6 | Schools should NOT allow transgender students to use restrooms that match with their gender identity. | 1 – Completely agree; 2 – Agree  3 – Disagree; 4 – Agree |
| Q18_4 | Some states have passed new laws that extend indecent exposure laws to include restrooms, locker and dressing rooms, or showers if the offender is a member of the opposite sex. Transgender people oppose these laws because they believe the law will affect their safety and security. Do you oppose or support this extension of indecent exposure laws? | 1 – Oppose strongly  2 – Oppose moderately  3 – Oppose a little  4 – Neither oppose nor support  5 – Support a little  6 – Support moderately  7 – Support strongly |
| Q18_5 | Here is a statement from opponents of these; how convincing it to you?  Transgender people oppose these laws because they believe the will affect their safety and security. | 1 – Not at all convincing  2 – Somewhat convincing  3 – Convincing  4 – Very convincing |
| Q18_6 | What is your opinion of such laws now, should indecent exposure laws be extended to include restrooms, locker and dressing rooms, or showers if the offender is a member of the opposite sex? | 1 – Oppose strongly  2 – Oppose moderately  3 – Oppose a little  4 – Neither oppose nor support  5 – Support a little  6 – Support moderately  7 – Support strongly |

**Supplementary Table 13. Question wordings documenting respondent traits.**

| **Trait** | **Wording** | **Response Set** |
| --- | --- | --- |
| Gay Friend/Family | Do you have a close friend or family member who is gay or lesbian? | 1 – Yes  2 – No |
| Transgender Friend/Family | Do you have a close friend or family member who is transgender? | 1 – Yes  2 – No |
| Racial Resentment (α=0.82) |  |  |
|  | Irish, Italians, Jewish and many other minorities overcame prejudice and worked their way up. Blacks should do the same without any special favors. | 1 – Agree strongly  2 – Agree somewhat  3 – Neither agree nor disagree  4 – Disagree somewhat  5 – Disagree strongly |
|  | Generations of slavery and discrimination have created condition that make it difficult for black to work their way out of the lower class. | 1 – Agree strongly  2 – Agree somewhat  3 – Neither agree nor disagree  4 – Disagree somewhat  5 – Disagree strongly |
|  | Over the past few years, black have gotten less than they deserve. | 1 – Agree strongly  2 – Agree somewhat  3 – Neither agree nor disagree  4 – Disagree somewhat  5 – Disagree strongly |
|  | It’s really a matter of some people not trying hard enough, if blacks would only try harder they could be just as well of as whites. | 1 – Agree strongly  2 – Agree somewhat  3 – Neither agree nor disagree  4 – Disagree somewhat  5 – Disagree strongly |
| Non-traditional Gender Roles (α=0.76) |  |  |
|  | The initiative in courtship should usually come from a man. | 1 – Strongly agree  2 – Agree  3 – Somewhat agree  4 – Neither agree nor disagree  5 – Somewhat disagree  6 - Disagree  7 – Strongly disagree |
|  | There are some professions and types of businesses that are more suitable for men than women. | 1 – Strongly agree  2 – Agree  3 – Somewhat agree  4 – Neither agree nor disagree  5 – Somewhat disagree  6 - Disagree  7 – Strongly disagree |
|  | It bothers me more to see a woman who is pushy than a man who is pushy. | 1 – Strongly agree  2 – Agree  3 – Somewhat agree  4 – Neither agree nor disagree  5 – Somewhat disagree  6 - Disagree  7 – Strongly disagree |
|  | It is disrespectful for a man to swear in the presence of a lady. | 1 – Strongly agree  2 – Agree  3 – Somewhat agree  4 – Neither agree nor disagree  5 – Somewhat disagree  6 - Disagree  7 – Strongly disagree |
| Gender Nonconformity Men (α=0.84) |  |  |
|  | I often wonder what it would be like to be a woman. | 1 – No  2 – Yes |
|  | In many ways, I feel more similar to women than to men. | 1 – No  2 – Yes |
|  | People think I should act more masculine than I do. | 1 – No  2 – Yes |
|  | I feel like a part of me is male and part of me is female. | 1 – No  2 – Yes |
|  | I often think I would rather be a woman. | 1 – No  2 – Yes |
|  | At times people in stores and restaurants have mistaken me for a woman. | 1 – No  2 – Yes |
| Gender Nonconformity Women (α=0.71) |  |  |
|  | I often wonder what it would be like to be a man. | 1 – No  2 – Yes |
|  | In many ways I feel more similar to men than to women. | 1 – No  2 – Yes |
|  | People think I should act more feminine than I do. | 1 – No  2 – Yes |
|  | I feel like part of me is female and part of me is male. | 1 – No  2 – Yes |
|  | I often think I would be a man. | 1 – No  2 – Yes |
|  | At times people in stores and restaurants have mistaken me for a man. | 1 – No  2 – Yes |
| Disgust (α=0.65) |  |  |
|  | I never let any part of my body touch the toilet seat in a public washroom. | 1 – Extremely like me  2 – Very much like me  3 – Somewhat like me  4 – Not like me |
|  | I probably would not go to my favorite restaurant if I found out that the cook had a cold. | 1 – Extremely like me  2 – Very much like me  3 – Somewhat like me  4 – Not like me |
|  | I use hand sanitizer on a daily basis. | 1 – Extremely like me  2 – Very much like me  3 – Somewhat like me  4 – Not like me |
| Authoritarianism (α=0.62) |  |  |
|  | Which one is more important for a child to have? | 1 – Independence  2 – Respect for Elders |
|  | Which one is more important for a child to have? | 1 – Curiosity  2 – Good Manners |
|  | Which one is more important for a child to have? | 1 – Obedience  2 – Self-Reliance |
|  | Which one is more important for a child to have? | 1 – Being Considerate  2 – Well-Behaved |
| Moral Non-traditionalism (α=0.67) |  |  |
|  | The world is always changing and we should adjust our view of moral to those changes. | 1 – Strongly agree  2 – Somewhat agree  3 – Neither agree nor disagree  4 – Somewhat disagree  5 – Strongly disagree |
|  | The newer lifestyles are contributing to the breakdown of our society. | 1 – Strongly agree  2 – Somewhat agree  3 – Neither agree nor disagree  4 – Somewhat disagree  5 – Strongly disagree |
|  | We should be more tolerant of people who choose to live according to their own moral standards, even if the yare different from out own. | 1 – Strongly agree  2 – Somewhat agree  3 – Neither agree nor disagree  4 – Somewhat disagree  5 – Strongly disagree |
|  | This country would have many fewer problems if there were more emphasis on traditional family ties. | 1 – Strongly agree  2 – Somewhat agree  3 – Neither agree nor disagree  4 – Somewhat disagree  5 – Strongly disagree |
| Partisanship (Dem. – Rep.) |  |  |
|  | In politics TODAY, do you consider yourself a Republican, Democrat, or independent, or what? | 1 – Republican  2 – Democrat  3 - Independent  4 – Other |
|  | Would you call yourself a strong [Republican/Democrat] or not very strong? | 1 – Strong  2 – Not very strong |
|  | Do you think of yourself as closer to the Democrat or Republican party? | 1 – Republican  2 – Democrat  3 – Neither |
| Race (White) | If you had to choose, what would you say is your race? Are you white, African-American, Asian, or some other race? | 1 – White/Caucasian  2 – Black/African-American  3 – Asian/Asian-American  4 – Some other race  5 – Mixed race |
| Gender (Female) | What is your gender? | 1 – Male  2 – Female |
| Age | What is your age? |  |
| Education (College Grad) | What was the last grade in school you completed? | 1 – 8th grade or less  2 – High school incomplete  3 – High school complete  4 – Some college, but no degree  5 – Associates degree  6 – College graduate/bachelors degree  7 – Postgraduate degree |

**Supplementary Table 14. Summary statistics of respondent traits.**

| Variable | Min | Max | *M* | *SD* |
| --- | --- | --- | --- | --- |
| Gay Friend/Family | 0 | 1 | 0.57 | 0.50 |
| Transgender Friend/Family | 0 | 1 | 0.16 | 0.36 |
| Racial Resentment | -2 | 2 | 0.06 | 1.08 |
| Non-traditional Gender Roles | 1 | 7 | 3.96 | 1.36 |
| Gender Nonconformity | 0 | 7 | 1.05 | 1.49 |
| Disgust | 1 | 4 | 2.68 | 0.88 |
| Authoritarianism | 0 | 4 | 2.21 | 1.33 |
| Moral Non-traditionalism | -2 | 2 | -0.02 | 0.83 |
| Partisanship (Dem. – Rep.) | 1 | 7 | 3.69 | 2.16 |
| White | 0 | 1 | 0.75 | 0.43 |
| Female | 0 | 1 | 0.54 | 0.50 |
| Age | 18 | 92 | 46.5 | 16.4 |
| College Grad | 0 | 1 | 0.40 | 0.49 |

## CONSORT Flow Chart

We provide the following CONSORT flow chart in Supplementary Figure 4 to describe the sample sizes of those who entered the survey, those who were excluded due to not meeting an attention check, and the number of respondents randomly assigned to treatment. Due to missingness in the post-test, we excluded 57 respondents after randomization. Missingness was not large within any treatment groups, and there was no systematic relationship between missingness and treatment assignment. Thus, we relied on a complete-case analysis.

**
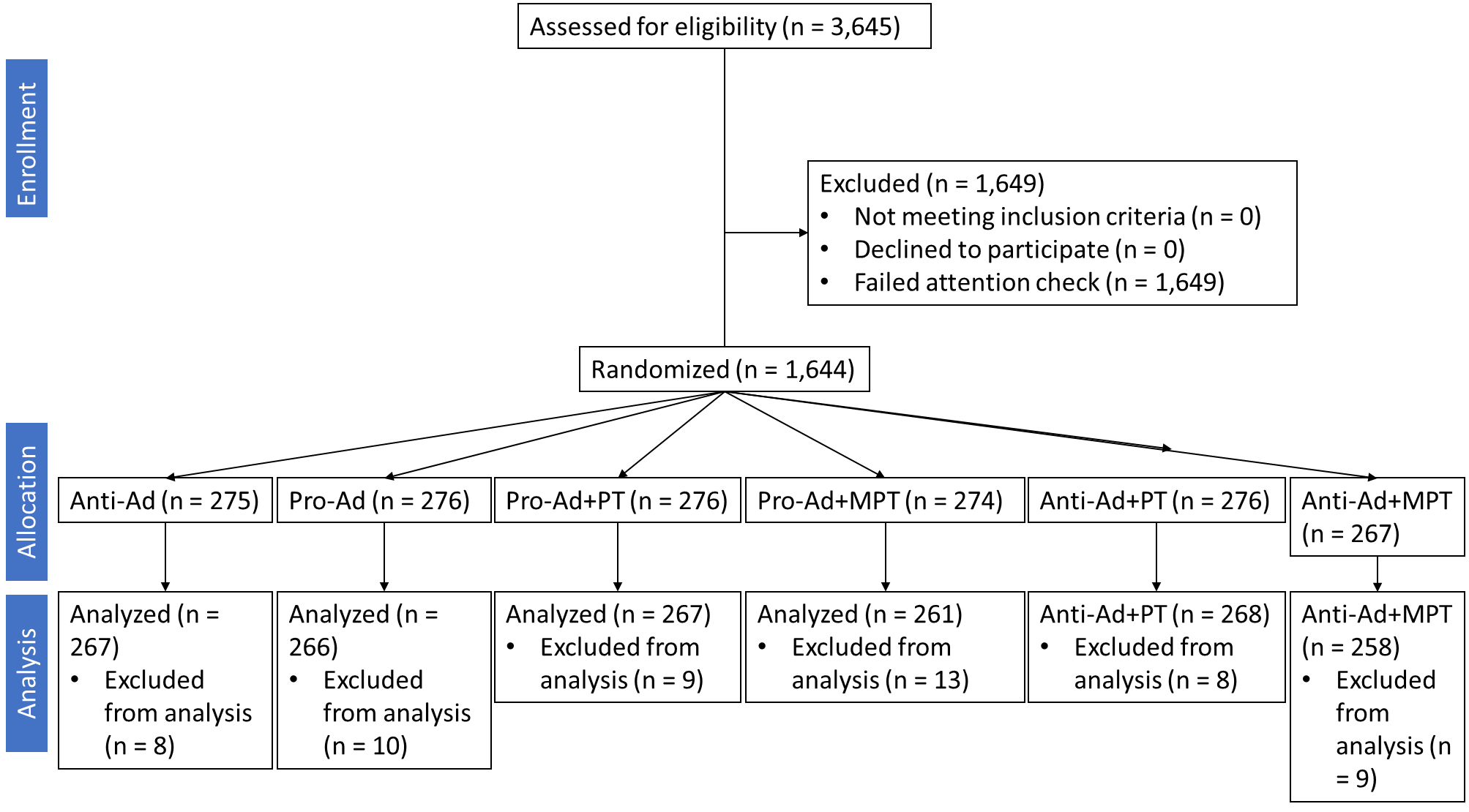
**

**Supplementary Figure 4. CONSORT Flow Chart.**

## Balance Checks

We report in Supplementary Table 15 balance tests between pre-treatment variables on whether or not respondents were assigned to the perspective taking, modified perspective taking or the control exercise. We report in Supplementary Table 16 balance test between pre-treatment variables on the ad conditions. If a chi-squared test statistic is reported, then the balance test was a test of independence. If an *F*-test statistics is reported, then a regression was fit to examine mean differences across treatment groups.

**Supplementary Table 15. Balance Tests on Narrative Exercise.**

| **Variable** | **Statistic** | **P-value** |
| --- | --- | --- |
| Gender | $\chi^{2}\left( 2 \right)=0.03$ | .99 |
| Partisanship (7-point) | $\chi^{2}\left( 12 \right)=18.92$ | .09 |
| Gender Conformity | *F* (2, 1584) = 0.20 | .82 |
| Racial Resentment | *F* (2, 1584) = 0.57 | .56 |
| Disgust Sensitivity | *F* (2, 1584) = 0.33 | .72 |
| Authoritarianism | *F* (2, 1584) = 0.76 | .47 |
| Traditionalism | *F* (2, 1584) = 1.70 | .18 |
| Gender Role Beliefs | *F* (2, 1584) = 1.37 | .25 |
| Egalitarianism | *F* (2, 1584) = 0.20 | .82 |

**Supplementary Table 16. Balance Tests on Advertisement Condition**

| **Variable** | **Statistic** | **P-value** |
| --- | --- | --- |
| Gender | $\chi^{2}\left( 1 \right)=0.33$ | .57 |
| Partisanship (7-point) | $\chi^{2}\left( 6 \right)=11.14$ | .08 |
| Gender Conformity | *F* (1, 1585) = 0.31 | .58 |
| Racial Resentment | *F* (1, 1585) = 1.19 | .28 |
| Disgust Sensitivity | *F* (1, 1585) = 1.73 | .19 |
| Authoritarianism | *F* (1, 1585) = 0.00 | .98 |
| Traditionalism | *F* (1, 1585) = 0.74 | .39 |
| Gender Role Beliefs | *F* (1, 1585) = 0.00 | .97 |
| Egalitarianism | *F* (1, 1585) = 0.12 | .72 |

## Length of Time Performing the Perspective-Taking Exercise

We assigned participants to engage with a traditional paragraph-style perspective taking exercise (PT), a modified perspective-taking that was designed to be more intense (MPT), and a control condition that still required the writing of a personal narrative. The MPT condition was designed to be more intensive. One way to assess this is to examine the amount of time participants spent in each of these conditions. Overall, participants spent an average of 85.4 seconds (*SD* = 149.7) performing the exercise with a minimum of 1.9 seconds and a maximum of 3,372.5 seconds. Here, we assess this relying on OLS regression, median regression, and a log-transformed regression because timing had a non-normal distribution. We find in Supplementary Table 17 that the MPT condition indeed did require more time of our participants, consistent with the expectation that the condition would be more intensive. We find that those who were in the MPT condition spend an additional 22 to 23 seconds on the exercise than those in the control condition. Those who were in the PT condition did not significantly differ from the control group. Supplementary Table 18 provides two-stage least squares results thus providing treatment effect estimates for compliers. According to the long-transformed model (Model 2), those who were assigned to PT or MPT spent more time on the exercise, with those in the MPT condition spending the most amount of time. Overall, these results are consistent with the expectation that a MPT exercise would induce greater effort than a traditional PT exercise.

**Supplementary Table 17. Regression Results on Length of Time in Each Condition.** ^ *p* < .010; * *p* < .05 (two-tailed).

|  | (1)  OLS | (2)  Median Regression | (3)  Log(Y) OLS |
| --- | --- | --- | --- |
| PT | 6.2 (9.0) | 0.96 (3.4) | 0.12 (0.06)^ |
| MPT | 22.4 (9.1)* | 23.0 (3.4)*** | 0.50 (0.06)*** |
| Intercept | 76.2 (6.4)*** | 44.0 (2.4)*** | 3.70 (0.04)*** |
| *N* | 1644 | 1644 | 1644 |
| *F* (*df*, *df*) | 3.26 (2, 1641)* | -- | 36.06 (2, 1641)*** |
| *R*^2^ | 0.004 | 0.02 | 0.04 |

**Supplementary Table 18. Two-Stage Regression Results on Length of Time in Each Condition.** ^ *p* < .010; * *p* < .05 (two-tailed).

|  | (1)  2SLS | (2)  Log(Y) 2SLS |
| --- | --- | --- |
| PT | 8.0 (11.7) | 0.15 (0.08)* |
| MPT | 28.8 (11.6)* | 0.64 (0.08)*** |
| Intercept | 76.2 (6.3)*** | 3.7 (0.04)*** |
| N | 1644 | 1644 |
| Wald-χ^2^ (*df*) | 6.61 (2)* | 78.24 (2)*** |
| *R*^2^ | 0.04 | 0.12 |

# References

Berinsky, A.J., Huber, G. A., & Lenz, G. S. (2012). Evaluating online labor markets for experimental research: Amazon. com's mechanical turk. *Political Analysis,* *20*(3), 351-68.

Mullinix, K. J., Leeper, T. J., Druckman, J. N., & Freese, J. (2015). The generalizability of survey experiments. *Journal of Experimental Political Science,* *2*(2), 109-38.
